# Dogs with Bilateral Medial Coronoid Disease Can Be Clinically Sound after Unilateral Arthroscopic Fragment Removal—Preliminary Study

**DOI:** 10.3390/ani13243803

**Published:** 2023-12-09

**Authors:** Sophia Seidler, Johannes Siedenburg, Michaela Rhode, Holger A. Volk, Oliver Harms

**Affiliations:** 1 Clinic for Small Animals, University of Veterinary Medicine Hannover Foundation, 30559 Hannover, Germany; 2 Clinic for Small Animals, 71640 Ludwigsburg, Germany; 3 Tierarztpraxis für Kleintierchirurgie, 58809 Neuenrade, Germany

**Keywords:** medial coronoid process disease, dogs, arthroscopy, conservative management, arthroscopic intervention, fragmented coronoid process, clinical outcome, radiographs

## Abstract

**Simple Summary:**

Medial coronoid disease of the elbow is a common lesion of the elbow in young and middle-aged dogs, which can cause pain and lameness. Various treatment options are available for this disease, all aimed at providing a pain-free life for the patient and minimizing potential consequential damage, such as osteoarthritis and cartilage damage. This study investigates the use of both conservative and surgical (arthroscopic) therapies for single dogs, with a focus on clinical outcomes (owner-based dog lifestyle and mobility evaluation), radiological changes, and gait patterns. The study has shown that arthroscopic treatment can improve gait patterns and enhance the overall quality of life for affected dogs, despite worsening radiological findings. Notably, diagnostic imaging often does not correlate with clinical outcomes. Computed tomographic findings, such as fragment size and the presence of dislocation, may influence the treatment outcomes. This was indicated in the group of conservatively treated limbs. However, further studies are needed to establish this relationship more precisely. In summary, a patient-specific evaluation, considering all available diagnostic options, should guide the appropriate treatment.

**Abstract:**

The purpose of this study was to assess the outcome of dogs with bilateral medial coronoid disease (MCD) treated with arthroscopic intervention for the clinically more severely affected side and conservative management for the contralateral side. The medical records of dogs with bilateral medial coronoid disease diagnosed using computed tomography (CT) and treated using arthroscopic intervention on one elbow and conservative management on the other elbow were retrospectively reviewed. The outcome evaluation included clinical re-examination; follow-up radiographic-visible osteophytic lesions; as well as sclerotic changes and Liverpool osteoarthritis in a dogs questionnaire. Data from 48 clinically affected elbow joints (24 dogs) with bilateral MCD diagnosed using CT were included. Every dog underwent arthroscopic intervention on the elbow joint, which was clinically more severely affected, and the other side was treated with conservative management. A fragment of the medial coronoid was diagnosed using CT in all elbows, whereas 19 elbows (39.4%) showed a dislocation of the fragment and the other 29 elbows (60.4%) did not. There are no findings regarding the radioulnar Incongruence. Initially, 86% of all radiographs had the same degree of osteophytes. At the time of follow-up, the arthroscopic-treated limbs had more severe radiological changes in comparison to the conservatively treated limbs. Lameness improved after arthroscopic therapy in walking. The conservative group showed a largely unchanged gait pattern. Radiological changes do not necessarily reflect the severity of clinical signs. Arthroscopic intervention showed an improvement of the clinical gait pattern, even though the radiographic changes worsened.

## 1. Introduction

Disease of the medial coronoid, as a component of elbow dysplasia complex, is one of the most frequently diagnosed heritable orthopedic disorders in dogs [1,2,3,4] and is also the most important cause of elbow lameness in large- and giant-breed dogs [4,5,6].

The majority of cases first present at an age around 5–12 months because of persistent forelimb lameness [3,4,7]. Some dogs present later in life (>6 years old), showing clinical manifestations of medial coronoid disease (MCD) without having shown signs of lameness previously [7,8]. Another noteworthy discovery, as reported by Moores et al., is that within their study cohort, exclusively composed of one specific breed, approximately 50% exhibited pathological abnormalities in the medial coronoid process (MCP) without displaying any observable clinical signs of lameness [9].

MCD encompasses both lesions of the articular cartilage and subchondral bone [10,11] and includes different disorders such as fissuring and fragmentation of the medial aspect of the coronoid process, displaced and non-displaced fragments, and chondromalacia-like lesions [5,12]. In clinical practice, radiographic examination remains the primary modality for screening elbow dysplasia (ED) despite variable sensitivity [12,13]. Challenges arise, however, due to the superimposition of the radial head in the medial coronoid process and the presence of osteophytes, which can hinder an accurate assessment of the MCP [14]. Additionally, the snug fit between the ulnar trochlear notch and the humeral condyle adds complexity to accurate diagnosis [15]. Frequently, a suspected diagnosis of medial coronoid disease (MCD) can only be inferred from secondary indicators such as osteophytes, obscuration of the cranial coronoid contour, and sclerosis of the ulnar notch [15,16]. Computed tomography (CT) findings are described as abnormalities in form of fragmentation, fissures, sclerosis or hypoattenuation, unusual morphology, and irregular radial incisures [9].

The etiology of MCD still remains uncertain and is suggested to have a polygenic and multifactorial origin [4,14]. The following theories are described in the literature: heritable disease, abnormalities of the underlying subchondral bone, and atypical mechanical loading [4]. Radioulnar incongruity also seems to play an important role [4,16]. Furthermore, additional environmental factors, including physical activity, dietary patterns, microtrauma, and mineral imbalance, cannot be discounted as potentially influential in the progression of MCD [4,13].

In the veterinary literature, the management of MCD is still controversial [4].

There are many treatment options which can be either surgical or conservative [4,17,18]. All of these treatments aim to restore normal function, alleviate pain, and slow the progression of osteoarthritis [16,17,19].

Michelsen compared in his literature review the outcome of different treatment options of MCD, including arthrotomy, arthroscopy, and medical management [7]. It was stated that arthroscopic fragment removal resulted in reduced morbidity and better outcomes than arthrotomy or conservative management when the disease was not advanced [7].

Other studies which compared conservative versus (vs.) arthroscopic treatment of MCD concluded that there was no difference in the outcome between these two treatment groups [7,17,18,20] and it ultimately led to osteoarthritis [8,18]. The elements affecting the outcome of dogs with MCD remain ambiguous [21].

Several factors such as the severity and duration of lameness at the time of presentation at the examining veterinarian, the extent of cartilage damage, osteoarthritis, and the specific type of lesions in the joint could collectively influence the outcome and prognosis [21].

The objective of our study was to evaluate the outcome of conservative management, consisting of a regulated exercise regimen, non-steroidal anti-inflammatory medication, and supportive exercises, as compared to arthroscopic Subtotal Coronoid Ostectomy (SCO) in a canine subject with bilateral MCD. The evaluation encompassed radiographs and a subjective gait analysis comparative before and after therapy, as well as owner questionnaires, focusing on the mobility and lifestyle of each dog after completion of the treatment. Additionally, we examined the progression of the non-surgically treated forelimb within the group by comparing the parameters derived from radiographs, CT, and gait analysis.

## 2. Materials and Methods

### 2.1. Inclusion and Exclusion Criteria

Clinical records from the Small Animal Teaching Hospital database at Tierärztliche Hochschule Hannover were reviewed for dogs diagnosed with bilateral MCD from February 2014 to December 2018. Prospective data were collected via follow-up examinations conducted from May 2019 to September 2019. Eligible dogs had a bilateral diagnosis of MCD, with the more clinically affected forelimb visible as a lameness, as well as pain and pathologic findings such as crepitation, external rotation of the forelimb, and joint swelling during the orthopedic examination, undergoing arthroscopic intervention, while the other forelimb received conservative treatment.

Retrospective data from radiographs and CT imaging were used for diagnosis, with fragmented medial coronoid processes in CT imaging serving as a decisive factor. The inclusion criteria involved documentation of radiographs, initial CT scans, arthroscopic surgery reports, and clinical examinations. Additional requirements included a subjective gait assessment, a completed owner questionnaire (including the Liverpool Osteoarthritis in Dogs [LOAD] Score) (Supplementary Materials [22,23,24]), and the availability of data at the follow-up examination.

For each dog, the collected data included name, date of birth, breed, sex, neuter status, weight, age at MCD diagnosis, and hospital identification number. The diagnostic findings from radiographs, modified International Elbow Working Group (IEWG) scores [25], Trochlear Notch Sclerosis (TNS) scores [13], and gait analysis results were also recorded.

Initial data from radiographs, CT imaging, and clinical examinations were sampled retrospectively. Prospective data collected during follow-up examinations were inclusive of radiographs, clinical examinations, gait analysis, and owner questionnaires. The study design was a non-blinded, observational study. Dogs that had died and those with concurrent elbow joint pathology such as ununited anconeal process, osteochondritis of the medial humeral condyle, and flexor tendon enthesiopathy or other orthopedic diseases were excluded.

### 2.2. Treatment Groups

Every dog had both arthroscopic intervention on one elbow and conservative intervention on the contralateral elbow for MCD. The decision on which limb was treated arthroscopically depended on the clinical examination and subjective gait assessment. The clinically more affected forelimb was treated arthroscopically with fragment removal. Arthroscopy was performed by the same veterinary surgeon. Conservative management and post-operative care of the arthroscopic intervention group included leash-walking the dog for 6 weeks, with non-steroidal anti-inflammatory drugs recommended over 14 days and supportive exercises like physiotherapy, aqua training, osteopathy, or electrotherapy. In some cases, feed additives to support the joint mechanism, for example, green-lipped mussel extract, devil’s claw, glucosamine, and chondroitin, were implemented as supportive measures (Table 1).

### 2.3. Radiographs

Radiographs including mediolateral flexed and craniocaudal views of each elbow joint were taken initially and post-surgery, respectively, and after conservative intervention during the follow-up examination. The period post-therapy refers to the time of clinical follow-up, which was a median of 32.5 months. The radiographs were evaluated for osteoarthritis according to the IEWG guidelines [25]. The scoring system was modified to focus solely on the size of the osteophytes as an indicator of arthrosis. This modification was necessary, as many radiographic images would otherwise have been consistently scored as 2 due to our diagnosis of MCD (Table 2).

In our adapted assessment, incongruence was not taken into account. Dogs with concurrent elbow joint pathologies, such as ununited anconeal process or osteochondritis dissecans (OCD), were already excluded, rendering these signals irrelevant for the evaluation.

**Figure 1:**
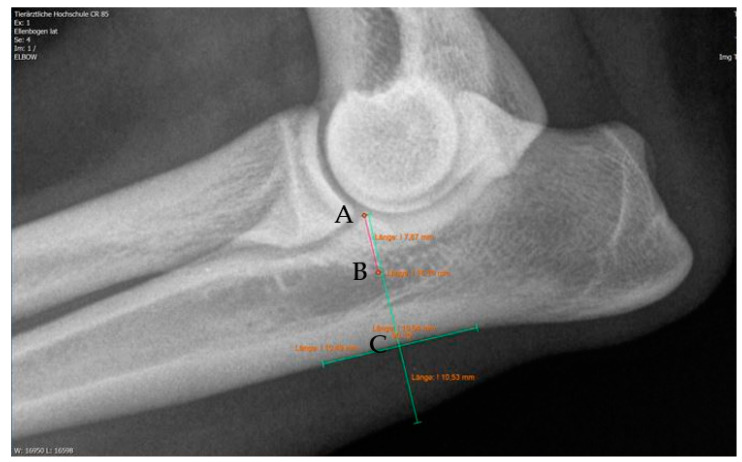
Measurement of the Trochlear Notch Sclerosis (TNS). Line AB: extent of sclerosis; line AC: ulnar depth 90° to caudal ulnar shaft → AB/AC = TNS [13].

Moreover, Trochlear Notch Sclerosis (TNS), a radiological term denoting increased bone radio-opacity in the ulnar trochlear notch region, was quantified. The measurements adhered to the methodology outlined by Draffan et al. [13], and the comprehensive TNS ratio of sclerosis to the ulnar depth was subsequently computed (Figure 1). All measurements were performed by the first author of the article.

### 2.4. Computed Tomography

CT imaging was conducted on both elbow joints of each dog utilizing a Philips Brilliance 64-slice scanner (Philips Medical Systems Technologies LTD, Haifa, Israel). The scanning parameters were adjusted based on body weight, with a predominant use of a 1 mm slice thickness, 0.579 pitch, 0.75 s rotation time, 120 kV, and 200 mAs/slice, employing a bone algorithm. The dogs were premedicated with Acepromazine at a dosage of 0.03 mg/kg (Tranquisol^®^ KH 0.5mg/mL, Cp-Pharma) and Levomethadone at 0.5 mg/kg (L-Polamivet 2.5/0.125 mg/mL, MDS Tiergesundheit, Merck & Co., Inc., Rahway, NJ, USA) to facilitate the CT procedure under anesthesia. Anesthesia was induced intravenously using Propofol at 4 mg/kg (PropoVet Multidose 10 mg/mL, Zoetis, Zoetis Inc., Parsippany, NJ, USA), and maintenance was achieved with Isoflurane in oxygen (Vetflurane^®^ 1000 mg/g, Virbac, Virbac Limited, Bury St. Edmunds, UK). All dogs were placed in a sternal recumbent position, with both forelimbs extended cranially at an angle ranging from 90° to 120°, following the method outlined by Shimizu et al. [14]. The parameters as described in Table 3 were assessed for each joint. All measurements were taken using an imaging software (Easy Image, 3.1.1, Veterinärmedizinisches Dienstleistungszentrum (VetZ) GmbH, Isernhagen, Germany, CoSi dental GmbH, Sigmaringen, Germany).

### 2.5. Clinical Examination

Each dog was clinically examined by the first and third author of the study between 6 and 65 months (median 32.5 months) after therapy in the small animal clinic of the University of Veterinary Medicine Hannover. The evaluation was not blinded. A subjective gait analysis was performed, evaluating the gait pattern of each dog in walking and trotting and rated based on the lameness score of Millis and Levine [26].

**Table 3:** Computed tomographic variables studied at the medial coronoid process (MCP) [16,27,28].

Type of Pathology Present at MCP	Type of Fragmentation of the MCP	Fragment Dislocation
Single fragment Multiple fragments Fissures Combination of lesions None of the above lesions	Fragment or fissure along the radial incisure of the ulna Fragmentation affecting the MCP at the apex Radial incisures–tip fragment or fissure (combination)	Yes No

### 2.6. Questionnaire

Owners were sent an email or physical letter summarizing the study aims and methodology with the questionnaire attached (Supplementary File S1: questionnaire). The questionnaire comprised information on the patient, details on current lifestyle habits, other diseases, medications, disease history of the elbow lameness, and the LOAD Score [22,23,24], which is a validated tool to assess canine articular disorders like osteoarthritis. The LOAD Score consisted of 13 questions, which were answered individually for each forelimb. The questions were answered by the owner of the dog and evaluated by the first author of the paper. A score between 0 and 4 was awarded per question, so that a total score between 0 and 52 could be achieved. The total score per limb was then interpreted as follows: Mild (0–10), Moderate (11–20), Severe (21–30), Extreme (31–52).

### 2.7. Statistical Analysis

Statistical analysis was performed using the software R version 3.6.0 (26 April 2019, R Foundation for Statistical Computing, Vienna, Austria). The following fundamental variables were recorded for the statistical calculations. The statistical analysis encompassed three datasets, incorporating details on the LOAD Score, analysis of the radiographs, and gait analysis (both in walking and trotting). Additionally, individual canine information such as age, breed, sex, weight, age at diagnosis, and intervention group (whether arthroscopy or conservative management was performed) was considered. Descriptive statistics were computed for all variables. Metric variables that exhibited a normal distribution were characterized by the mean value (MW) and standard deviation (SD) and compared using either a *t* test or Kruskal–Wallis test. Conversely, skewed variables were described using the median and interquartile range (IQR), and their equal positional distribution was assessed using a nonparametric test, either Wilcoxon’s rank-sum test or the Kruskal–Wallis test. Categorical variables were outlined using absolute (N) and relative frequencies (%) and subjected to comparison via the χ^2^ independence test. A significance level of <0.05 was deemed as statistically significant. 

## 3. Results

Altogether, out of the 55 potential participants in the study, only 24 dogs met the inclusion criteria. This was due to the fact that 31 dogs had either died, or their owners chose not to participate in the study for various reasons. The most common breed was cross-breed dogs (*n* = 9, 37.5%), followed by Labrador Retriever (*n* = 6, 25%). The individual breeds are shown in Table 4. The bodyweight of the 24 examined dogs ranged from 10.5 to 68.5 kg (median: 35.2 kg). The age at diagnosis was between 5 and 94 months (median: 37.4 months ± 29.2) and the age at time of the follow-up examination ranged from 20 to 151 months (median: 70.9 months). The gender distribution in the study population was as follows: 13 entire males, 5 neutered males, 3 entire females, and 3 spayed females. One dog showed no visible lameness during our clinical examination, but was presented in the hospital because of the lameness of one forelimb, which was finally treated arthroscopically. The remaining dogs were lame on the thoracic limb, which was later treated arthroscopically.

### 3.1. Radiographs

To assess the starting situation, the modified IEWG score of both therapy groups was compared before the beginning of the therapy. Among all the included patients, 12 dogs had a modified IEWG score of 0 in both the arthroscopic and conservative group. Three dogs had a modified IEWG score of 0 while the other group had a score of 1 and vice versa. Three times, the arthroscopic therapy group showed a higher modified IEWG value compared to the conservative group. In two cases, there was a difference of two and once a difference of one “score point”. To summarize, 85.6% of the dogs had the same degree of osteophytes as they did initially (Table 5).

At the time of the follow-up examination, the conservative and arthroscopic therapy groups were again evaluated in comparison. The following results were found: 5 dogs had the same modified IEWG score, 16 dogs from the arthroscopic group had a higher modified IEWG score, whereas only 1 dog from the conservative group had a higher modified IEWG score compared to the arthroscopic group (Table 6). Subsequently, each therapy group was compared individually before and after therapy. Regarding the arthroscopic group, 20% of the dogs had the same score after the arthroscopic intervention based on the modified IEWG score compared to the initial examination. None of the dogs improved post-surgery. Most of the dogs (80%) showed a worse modified IEWG score after the arthroscopy. The development of the conservative group was as follows: At the time of the X-ray examinations following the completion of conservative therapy, 58% of the dogs exhibited an unchanged modified IEWG score. In contrast, the remaining 42% of the forelimbs treated conservatively displayed a deterioration in their modified IEWG score when compared to the initial assessments.

Viewing the TNS, both intra- and inter-individual calculations were carried out. Hence, for the intra-individual observations, the difference was calculated for each dog and the median was calculated from these results. Paired (intra-individual), the TNS had increased by 0.04 mm for the median of the arthroscopic-treated elbows. Without considering the intra-individual differences, the median TNS value for the arthroscopic-treated elbows was 0.47 mm pre-operatively and 0.53 mm post-operatively. The *p*-value showed statistical significance (*p* = 0.022) for the TNS value comparing the pre- vs. post-therapy conditions for this group. For the conservatively treated elbows, the median TNS did not change when the intra-individual differences were formed (comparing before and after therapy; median = 0), but without considering the intra-individual differences, the median TNS value pre-operatively was 0.45 mm and post-operatively 0.50 mm. There was no significant difference in the TNS value of the elbows treated conservatively (*p* = 0.228). If the TNS value increased intra-individually, the enlargement was quite more evident compared to the elbows, with a reduction in the TNS value (maximum reduction in the TNS value is stratified after conservative or arthroscopic treatment at 0.07 mm or 0.08 mm, respectively, whereas the maximum enlargement is 0.12 mm or 0.20 mm, respectively).

### 3.2. Computed Tomography

Initially, a CT scan was obtained for each forelimb of the dogs, yielding a total of 48 scans. These scans were assessed with regard to pathology, the specific type of fragmented coronoid process (FCP), and dislocation (see Table 3). Moreover, the size of the fragment was computed for 47 elbow joints. A single fragment of the medial coronoid process was the most common pathology, occurring in 75–79% of cases in both the arthroscopic group and the conservative group. The remaining pathologies were relatively evenly distributed between the two groups (Table 7). Dislocation was present in 54.2% of the arthroscopically treated forelimbs, while the remaining 45.8% showed no dislocation. In the conservative therapy group, the fragment was dislocated only in 25%, while the remaining 75% of the fragments were not dislocated. The size of the fragments was measured in cm^2^. The median size of the arthroscopic-treated forelimbs was 0.185 cm^2^, while in the conservative group, it was 0.124 cm^2^.

### 3.3. Subjective Gait Analysis

The degree of lameness was recorded for each forelimb individually both in walking and trotting as well as for the two times of measurements at the initial examination and at the follow-up examination, a median of 32.5 months after therapy. In total, 184 values of 24 dogs were available for the gait analysis. There were eight missing values of the degree of lameness before therapy began due to incorrect documentation. To illustrate the success or failure of the therapy, the gait pattern of each therapy group was compared individually pre- to post-therapy, both in walking and trotting (Table 8, Table 9, Table 10 and Table 11). In walking, 83.3% of the conservatively treated dogs did not change their degree of lameness and 16.7% worsened. In comparison, 30% of dogs from the arthroscopic intervention group did not change in their degree of lameness, 50% improved, and 20% of the dogs deteriorated. Looking at the degree of lameness in trotting of the conservatively treated elbows, 87.5% of the dogs showed no change in their degree of lameness. The remaining 12.5% of this group deteriorated. In the arthroscopic-treated group, the degree of lameness in trotting did not change in 35% of the dogs, 30% had improved, and 35% had worsened.

### 3.4. Questionnaire

The questionnaires were filled out completely and sent back to us by the dog owners after therapy during the period of follow-up examinations. One questionnaire was missing, as it was not sent to us by the owner. Regarding the inter-individual analysis, the median of the achieved LOAD Score was calculated for each group (arthroscopic and conservative). The median score of the conservatively treated limbs was 9 points (mild), whereas the arthroscopic group achieved 10 points (mild). Thus, the difference regarding the median between the two groups was one point. In addition, the mean value was calculated: the conservative group scored 9.6 points (mild) and the arthroscopic group 13 points (moderate). When the intra-individual difference is considered, it can be seen that dogs from the arthroscopic group scored 2 points more in the median compared to the conservative group. Dogs from the arthroscopic-treated group had a significantly higher LOAD Score compared to the conservatively treated dogs (*p* = 0.003). The smallest difference between the two treatment groups is 4 points (smaller LOAD Score of the arthroscopic treated leg in comparison to the conservatively treated leg) and the largest difference is 15 points (larger LOAD Score of the arthroscopic-treated leg in comparison to the conservatively treated leg). In summary, it can be said that the arthroscopic-treated leg has a higher or equal LOAD Score in 75% compared to the conservatively treated leg.

Finally, a correlation analysis was carried out between the following measurement parameters: X-rays (modified IEWG, TNS), LOAD Score, and degree of lameness (Table 12). Looking at the correlation between the degree of lameness and the modified IEWG score, it can be seen that for both the conservative and arthroscopic therapy group, all dogs with a modified IEWG of 3 showed no lameness in walking at the time of the initial examination. Dogs with a modified IEWG score of 2 had an evenly distributed lameness score of 0 or 1 in the gait pattern at the first presentation. At trotting, a modified IEWG score of 2 or 3 was evenly distributed with a lameness score of 0 or 1 initially. Here, the two treatment groups were considered together. At the time of the follow-up examination, the correlation analysis did not show a consistent picture, neither in walking nor in trotting. A slightly positive correlation can be seen at the time of the follow-up in walking between the degree of lameness and the TNS. Otherwise, this analysis does not show a uniform distribution. Regarding the correlation between the LOAD Score and the modified IEWG at the follow-up for both therapy groups, on average, a higher LOAD Score was related to an increase in the modified IEWG. A slightly positive correlation (0.2) can be seen between the two characteristics of the LOAD Score and TNS.

To evaluate the different developments within the conservative therapy group, we compared the parameters of the radiographs, CT, and the gait analysis.

In the post-therapy radiographs, 8 out of 20 dogs from the conservative therapy group showed a worse modified IEWG score. Six of these dogs had a larger fragment in the CT than the mean value (0.12 cm^2^) and 50% of these legs showed a dislocation of the fragment.

Regarding the CT results, we can describe that dogs with a dislocated fragment (*n* = 6) showed more often a fragment size above average (*n* = 4) and a deterioration in the modified IEWG score (*n* = 4). Also, the dogs with a fragment of above-average size (*n* = 9) had a worse modified IEWG Score in the post-therapy examination (*n* = 6).

## 4. Discussion

The purpose of our study was to evaluate and describe the outcomes of dogs diagnosed with bilateral MCD treated with both arthroscopic and conservative interventions. The evaluation involved radiographs, subjective gait analysis, and owner questionnaires (LOAD Score). The arthroscopic intervention group showed a deterioration in the modified IEWG score, reflecting radiological osteophytic formations at the time of follow-up compared to the conservative therapy group. Specifically, 80% of the arthroscopic group showed deterioration in this aspect, while in the conservative group, it was approximately half (42%). This could be due to several reasons, for instance, the manipulation of the joint surface during arthroscopy, as already described in the literature [20,29], or differences in the initial condition of the joints. Also, the median TNS of the arthroscopic-treated group showed a higher TNS value of 0.06 mm post-operatively and was statistically assessed as significantly different. It should be questioned whether this very small difference of 0.06 mm is clinically relevant, as this parameter is described as a radiographic sign indicative of increased bone hardening, and further diagnostics are recommended in order to assess the severity of disease [13].

Owners reported an improvement in the gait pattern of the arthroscopic-treated forelimb after arthroscopic treatment. However, the assessment of the gait pattern gave different results: in walking, 50% of the dogs from the arthroscopic group showed an improvement, whereas only 30% exhibited such a positive development in trotting. The gait pattern of the dogs from the conservatively treated group remained largely unchanged, as assessed by the examining veterinarian during the follow-up examination (in walking: 83.3% unchanged, 16.7% worsened; in trotting: 87.5% unchanged, 12.5% worsened).

Concerning the LOAD Score provided by the owners, it is evident that, in their perception, there was only a slight difference between the two treated forelimbs after therapy. This assessment must be viewed critically since there are no initial questionnaire data. The forelimb clinically deemed worse was treated using arthroscopy, potentially introducing bias into our results. A less favorable starting condition of the arthroscopic therapy group could be assumed, influencing the outcome. Given that existing studies typically compare the progression of MCD treated with conservative vs. arthroscopic management in separate study groups [17,20], our study aimed to describe the progression and investigate the response to the two different therapy options in single dogs, avoiding bias due to genetics or physical exercise regimens. When interpreting the results, one should carefully balance the clinical picture—specifically, the quality of life—against the radiological signs of osteoarthritis and pathological findings.

Both the values of the modified IEWG score and the gait pattern of the arthroscopic and the conservative therapy groups were compared: almost 86% of the dogs had the same modified IEWG score before starting therapy. The conservatively treated limbs did not show any apparent lameness, but this could have been masked by the more affected side, which was treated arthroscopically. Given the absence of an objective gait analysis utilizing plate measurement, particularly ground reaction forces, there arises a query regarding the potential bias in the clinical gait analysis conducted in this study. It is noteworthy that a bilateral diagnosis of MCD was established using CT, and the existing literature has previously noted instances where dogs exhibited unilateral lameness despite a bilateral diagnosis of MCD. This complexity in the disease presentation underscores the intermittent or constant nature of clinical signs in affected dogs [17,18]. In addition, the evaluator of the lameness score was not blinded. Furthermore, it should be recognized that radiographic findings may not correlate with the severity of clinical signs [18,30,31]. This pattern is affirmed within the study when examining the correlation between the degree of lameness and the modified IEWG score. Notably, dogs with a modified IEWG score of 3 exhibited no lameness during walking in the initial examination. When trotting, dogs with a modified IEWG score of 3 displayed a uniform distribution of lameness scores between grades 0 and 1. The analysis of the correlation between the degree of lameness and TNS revealed a slight positive correlation during the follow-up when walking, regardless of the therapeutic method. It is important to acknowledge that a significant number of dogs showed no lameness, resulting in very limited case numbers for lameness grades 1–3. The LOAD Score exhibited a slight positive correlation with the modified IEWG for both therapy groups and the TNS. However, it is worth noting that the TNS can vary due to factors such as patient positioning, radiographic quality, and observer measurement capabilities [13].

As the results when comparing the arthroscopic vs. the conservative group could by biased, we also wanted to compare the different findings within the conservative group. Is there any difference between the conservatively treated forelimbs which can explain a better or worse outcome?

In the descriptive analysis, we found out that the modified IEWG score deteriorated more often when the fragment was larger than the mean value. Furthermore, a dislocated fragment was more common in combination with a larger fragment and also a deterioration in the modified IEWG score. We could not see any correlation between the gait pattern and the diagnostic imaging (radiographs, CT).

The median age at diagnosis for the dogs was 37.4 months, surpassing the average, while the median weight of 32.5 kg aligned with findings from comparable studies. Crossbreed dogs, Labrador Retrievers, and male dogs exhibited over-representation, a trend consistent with observations in other studies [13,14,17]. Body weight was only documented in kg and not as a body condition score (BCS), so there was no exact measure of actual obesity. Another limitation of the study is the small number of dogs involved, and the duration between the therapy and follow-up examinations ranged from 5 to 65 months. In addition, there were no questionnaires available before the start of therapy. A final limitation is that there was no uniform time period for follow-up examinations, and a new CT scan at the time of follow-up would probably have contributed to a better assessment of the treatment outcome. Unfortunately, this was not feasible due to financial constraints.

## 5. Conclusions

The results of this study should be considered preliminary, as it was a non-controlled study. Despite the radiological deterioration of the elbow in dogs undergoing arthroscopy, the dogs did appear to improve clinically. However, there is no evidence to suggest that conservative therapy for the surgically treated forelimbs would not have achieved similar results. It is important to bear in mind that arthroscopy, despite being a minimally invasive technique, is still an invasive procedure.

It could be argued that the radiographic findings do not provide a direct and definitive link to the gait pattern and the level of pain.

Regarding the forelimbs treated conservatively, there are indications that factors such as the size and position of the fragment, including the possibility of dislocation, could impact the therapy’s outcome. However, it is worth noting that the decision on whether and what type of conservative treatment was administered to the animals depended on the owner’s choice.

Our study has demonstrated that there is no clear-cut answer when it comes to the factors and variables affecting the clinical presentation, treatment, and outcomes of medial coronoid disease. Prospective, randomized, controlled studies that include objective gait analysis using ground reaction forces, CT examinations at follow-up, and long-term follow-ups at standardized intervals are warranted. Conducting a comparative analysis of the cartilage condition before and after therapy using the modified Outerbridge Score [11] would provide valuable insights into the efficacy of the chosen therapeutic approach and its impact on the cartilage. There is evidence that a large fragment may potentially be associated with an increase in the modified International Elbow Working Group (IEWG) score, but this requires further statistical investigation and confirmation in subsequent studies.

Since, in this study, the clinically less affected side did not deteriorate in most cases, this raises questions about when MCD should be surgically addressed. A general consensus regarding therapy suggests that it is advisable not to rely solely on imaging diagnostics but to consider the patient and their owner’s input as part of the overall picture for determining the course of treatment.

## Figures and Tables

**Table 1:** Additional therapies and feed additives.

Dog Nr.	Rehabilitation Conservative	Rehabilitation Surgical	Feed Additives
1	None	None	Green-lipped mussel extract
2	Physiotherapy	Physiotherapy	Glucosamine, chondroitin
3	None	None	Green-lipped mussel extract
4	Physiotherapy, aqua training	Physiotherapy, aqua training	Glucosamine, chondroitin, omega-3-fatty acids
5	None	None	-
6	None	None	Green-lipped mussel extract, glucosamine, hyaluronan
7	None	None	-
8	None	None	-
9	Aqua training, osteopathy	Aqua training, osteopathy	Green-lipped mussel extract
10	None	None	-
11	None	None	-
12	None	None	-
13	None	None	Calcium, selenium, vitamin D3, green-lipped mussel extract
14	Physiotherapy	Physiotherapy	-
15	Aqua training	Aqua training	Green-lipped mussel extract, hyaluronan
16	Aqua training	Aqua training	Glucosamine, chondroitin, green-lipped mussel extract
17	Physiotherapy, aqua training ElectrotherapyOsteopathy, hyaluron injection intra-articular	Physiotherapy, aqua training Electrotherapy, osteopathy	Methylsulfonylmethan, chondroitin, glucosamine, green-lipped mussel extract
18	None	None	Omega-3-fatty acids, glucosamine, chondroitin, hyaluronan
19	None	None	Glucosamine, chondroitin, hyaluronan
20	Physiotherapy, aqua training	Physiotherapy, aqua training	Green-lipped mussel extract, omega-3-fatty acids, devil’s claw,
21	Physiotherapy	Physiotherapy	hyaluronan, omega-3-fatty acids
22	None	None	-
23	Physiotherapy, aqua training	Physiotherapy, aqua training	-
24	Physiotherapy	Physiotherapy	Feed additives unknown

Legend: The therapy recommendations for the analgesic and antiphlogistic medication—non-steroidal anti-inflammatory drugs (NSAID) and exercise management were as followed—NSAID: recommended over 14 days, partially variable by individual as required; exercise management: all dogs had restricted lead walk for 6 weeks; only additional treatment is listed.

**Table 2:** Modified elbow dysplasia scoring.

Modified Elbow Dysplasia Scoring	Radiographic Findings
0	Normal elbow joint: No evidence of sclerosis or arthrosis
1	Mild arthrosis: Presence of osteophytes < 2 mm high. Minor sclerosis of the base of the coronoid processes.
2	Moderate arthrosis: Presence of osteophytes 2–5 mm high. Obvious sclerosis of the base of the coronoid processes
3	Severe arthrosis: Presence of osteophytes of >5 mm high).
**Modified Elbow Dysplasia Scoring**	**Radiographs**
0	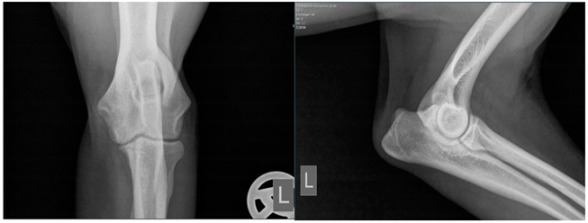
1	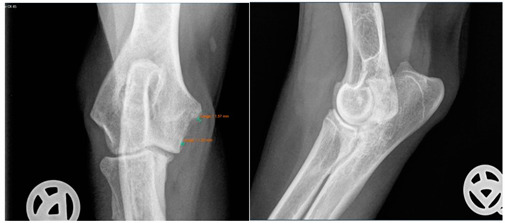
2	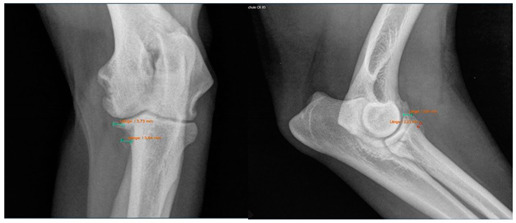
3	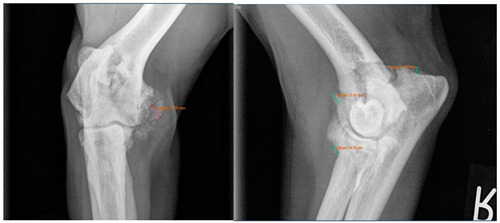

**Table 4:** Number (percentage) of breed dogs with bilateral medial coronoid disease.

Breed	No. of Dogs (%)
Airdale Terrier	1 (4.2%)
American Stafford Terrier	1 (4.2%)
Bullmastiff	1 (4.2%)
Ciobanese Mioritic	1 (4.2%)
German Shepard	1 (4.2%)
Golden Retriever	1 (4.2%)
Labrador Retriever	6 (25%)
Mastin de los Pirineo	1 (4.2%)
Mixed breed	9 (37.5%)
Rhodesian Ridgeback	1 (4.2%)
Rottweiler	1 (4.2%)

**Table 5:** Contingency table comparing modified IEWG before therapy.

Surgical			Conservative		
	**0**	**1**	**2**	**3**	**Total**
**0**	12 (57.1%)	3 (14.3%)	0 (0%)	0 (0%)	15 (71.4%)
**1**	3 (14.3%)	0 (0%)	0 (0%)	0 (0%)	3 (14.3%)
**2**	1 (4.8%)	0 (0%)	0 (0%)	0 (0%)	1 (4.8%)
**3**	0 (0%)	1 (4.8%)	1 (4.8%)	0 (0%)	2 (9.5%)
**Total**	16 (76.2%)	4 (19.0%)	1 (4.8%)	0 (0%)	21 (100%)

**Table 6:** Contingency table comparing modified IEWG after therapy.

Surgical			Conservative		
	**0**	**1**	**2**	**3**	**Total**
**0**	2 (9.1%)	0 (0%)	0 (0%)	0 (0%)	2 (9.1%)
**1**	1 (4.5%)	0 (0%)	1 (4.5%)	0 (0%)	2 (9.1%)
**2**	4 (18.2%)	2 (9.1%)	1 (4.5%)	0 (0%)	7 (31.8%)
**3**	2 (9.1%)	2 (9.1%)	5 (22.7%)	2 (9.1%)	11 (50%)
**Total**	9 (40.9%)	4 (18.2%)	7 (31.8%)	2 (9.1%)	22 (100%)

**Table 7:** General description of the lesions (CT variables).

Variable	Arthroscopic Group (No. (%))	Conservative Group (No. (%))	*p*-Value
**Pathology**	24 (100%)	24 (100%)	0.531
Fissure	1 (4.2)	2 (8.3)	
Combination of lesions	1 (4.2)	1 (2.4)	
Multiple fragments	4 (16.7)	1 (4.2)	
None of the above lesions	0 (0.0)	1 (4.2)	
Single fragment	18 (75.0)	19 (79.2)	
**Type of fragmented MCP**	24 (100%)	24 (100%)	0.629
Radial incisure–tip fragment or fissure (combination)	5 (20.8)	3 (12.5)	
Radial incisure fragment or fissure	8 (33.3)	7 (29.2)	
Tip fragment or fissure	11 (45.8)	14 (58.3)	
**Dislocation**	24 (100%)	24 (100%)	0.077
Yes	13 (54.2)	6 (25.0)	
No	11 (45.8)	18 (75.0)	
**Size of the Fragment**	24 (100%)	23 (100%)	0.660
Space/surface (cm^2^)	0.185	0.124	

**Table 8:** Results of gait analysis—subjective gait analysis in walking of dogs with bilateral medial coronoid disease treated conservatively.

Initial Examination	Follow-Up Examination				
**Lameness Degree**	**Lameness Degree**				
	**0 1 2 3 Total**				
**0**	20 (100%)	3 (12.5%)	1 (4.2%)	0 (0.0%)	24 (100%)
**1**	0 (0.0%)	0 (0.0%)	0 (0.0%)	0 (0.0%)	0 (0.0%)
**2**	0 (0.0%)	0 (0.0%)	0 (0.0%)	0 (0.0%)	0 (0.0%)
**3**	0 (0.0%)	0 (0.0%)	0 (0.0%)	0 (0.0%)	0 (0.0%)
**Total**	20 (83.3%)	3 (12.5%)	1 (4.2%)	0 (0.0%)	24 (100%)

**Table 9:** Results of gait analysis—subjective gait analysis in walking of dogs with bilateral medial coronoid disease treated arthroscopically.

Initial Examination	Follow-Up Examination				
**Lameness Degree**	**Lameness Degree**				
	**0 1 2 3 Total**				
**0**	3 (15.0%)	0 (0.0%)	0 (0.0%)	1 (5.0%)	4 (20.0%)
**1**	4 (20.0%)	2 (10.0%)	3 (15.0%)	0 (0.0%)	9 (45.0%)
**2**	3 (15.0%)	2 (10.0%)	1 (5.0%)	0 (0.0%)	6 (35.0%)
**3**	1 (5.0%)	0 (0.0%)	0 (0.0%)	0 (0.0%)	1 (5.0%)
**Total**	11 (55.0%)	4 (20.0%)	4 (20.0%)	1 (5.0%)	20 (100%)

**Table 10:** Results of gait analysis—subjective gait analysis in trotting of dogs with bilateral medial coronoid disease treated conservatively.

Initial Examination	Follow-Up Examination				
**Lameness Degree**	**Lameness Degree**				
	**0 1 2 3 Total**				
**0**	21 (87.5%)	0 (0.0%)	2 (8.3%)	1 (4.2%)	24 (100%)
**1**	0 (0.0%)	0 (0.0%)	0 (0.0%)	0 (0.0%)	0 (0.0%)
**2**	0 (0.0%)	0 (0.0%)	0 (0.0%)	0 (0.0%)	0 (0.0%)
**3**	0 (0.0%)	0 (0.0%)	0 (0.0%)	0 (0.0%)	0 (0.0%)
**Total**	21 (87.5%)	0 (0.0%)	2 (8.3%)	1 (4.2%)	24 (100%)

**Table 11:** Results of gait analysis—subjective gait analysis in trotting of dogs with bilateral medial coronoid disease treated arthroscopically.

Initial Examination	Follow-Up Examination				
**Lameness Degree**	**Lameness Degree**				
	**0 1 2 3 Total**				
**0**	3 (15.0%)	1 (5.0%)	1 (5.0%)	0 (0.0%)	5 (25.0%)
**1**	0 (0.0%)	2 (10.0%)	0 (0.0%)	2 (10.0%)	4 (20.0%)
**2**	2 (10.0%)	2 (10.0%)	2 (10.0%)	3 (15.0%)	9 (45.0%)
**3**	2 (10.0%)	0 (0.0%)	0 (0.0%)	0 (0.0%)	2 (10.0%)
**Total**	7 (35.0%)	5 (25.0%)	3 (15.0%)	5 (25.0%)	20 (100%)

**Table 12:** Descriptive statistic of the scores stratified by therapy.

	Surgical			Conservative			
Variable	N	NAs	Metrics	N	NAs	Metrics	*p*-Value
**Lameness degree N (%)**	88	8		96	0		
0			31 (35.3)			89 (92.7)	<0.001
1			25 (28.4)			3 (3.1)	
2			22 (25)			3 (3.1)	
3			10 (11.4)			1 (1.0)	
**Modified IEWG N (%)**	44	4		43	5		
0			17 (38.6)			25 (58.1)	0.020
1			6 (13.6)			8 (18.6)	
2			8 (18.2)			8 (18.6)	
3			13 (29.5)			2 (4.7)	
**TNS;** **MW (SD)**	42	6	0.510 (0.097)	41	7	0.478 (0.069)	0.085
**LOAD Score; MW (SD)**	23	1	13.000 (8.410)	23	1	9.609 (5.639)	0.116

MW = mean value, SD = standard deviation; *p*-values are employed to assess independence in the case of categorical variables or to test for differences in location when dealing with numerical variables.

## Data Availability

Data are contained within the article and Supplementary Materials.

## Supplementary Materials

The following supporting information can be downloaded at https://www.mdpi.com/article/10.3390/ani13243803/s1: All data generated or analysed during this study are included in this published article and its supplementary information files.
