# Supplementary material for: Dogs with Bilateral Medial Coronoid Disease Can Be Clinically Sound after Unilateral Arthroscopic Fragment Removal—Preliminary Study
Source: Animals (Basel). 2023 Dec 9;13(24):3803. doi: 10.3390/ani13243803 (PMC10741036; doi:10.3390/ani13243803)

Provided figure with sample radiographic images of each of the four grades of elbow dysplasia included in Tables 2 + 3

Table 2. - Modified Elbow Dysplasia Scoring

Modified Elbow Dysplasia Scoring	Radiographic Findings
0	Normal elbow joint: No evidence of sclerosis or arthrosis
1	Mild arthrosis: Presence of osteophytes < 2 mm high. Minor sclerosis of the base of the coronoid processes.
2	Moderate arthrosis: Presence of osteophytes of 2 - 5 mm high. Obvious sclerosis of the base of the coronoid processes
3	Severe arthrosis: Presence of osteophytes of > 5 mm high).

Modified Elbow Dysplasia Scoring - Grade 0

Radiograph of a left elbow in a mediolateral flexed view
Grade 0: Normal elbow joint - No evidence of sclerosis or arthrosis

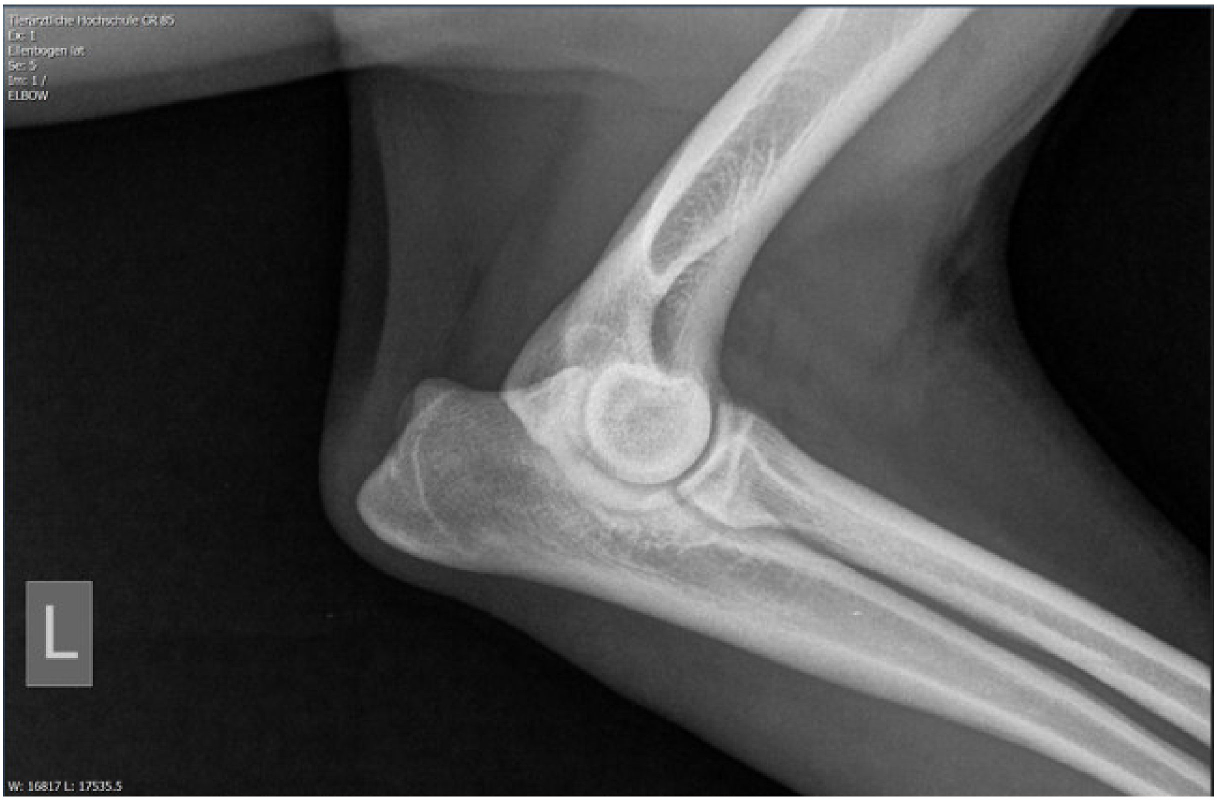

Radiograph of a left elbow in a craniocaudal view
Grade 0: Normal elbow joint - No evidence of sclerosis or arthrosis

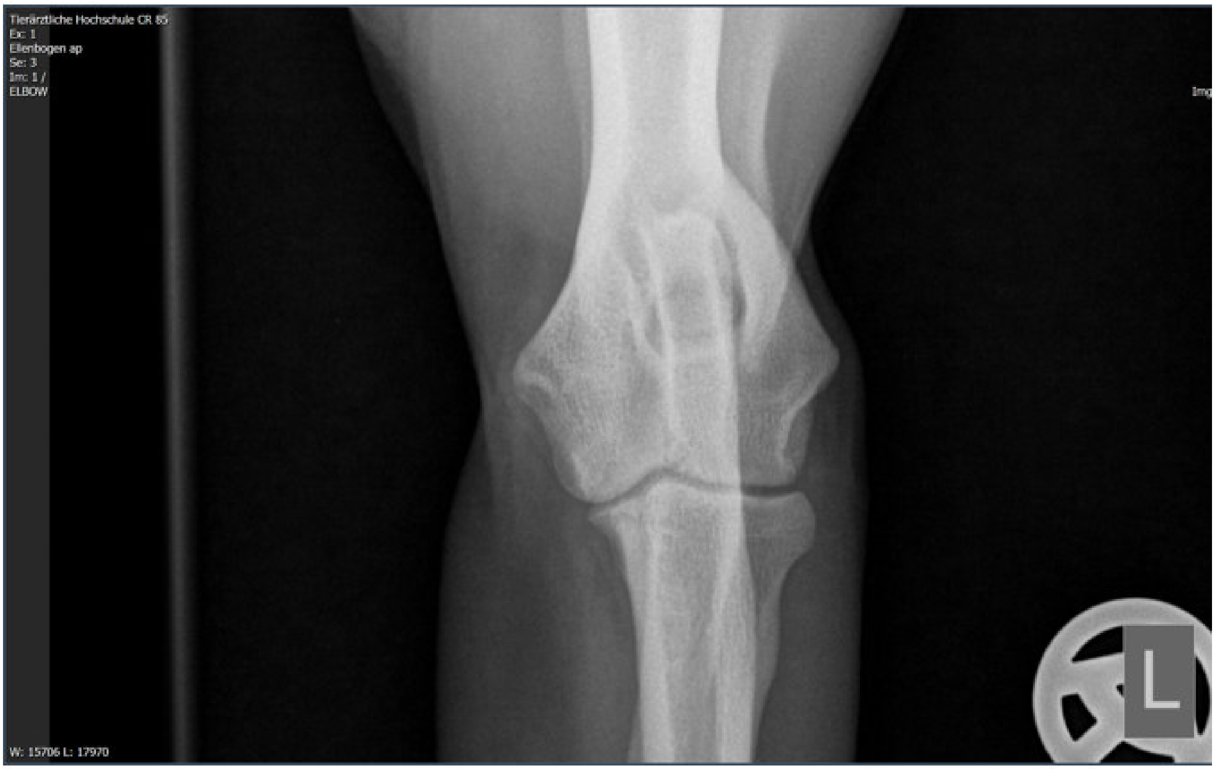

Modified Elbow Dysplasia Scoring - Grade 1

Radiograph of a right elbow in a mediolateral flexed view
Grade 1: Mild arthrosis - Presence of osteophytes < 2 mm high. Minor sclerosis of the base of the coronoid processes

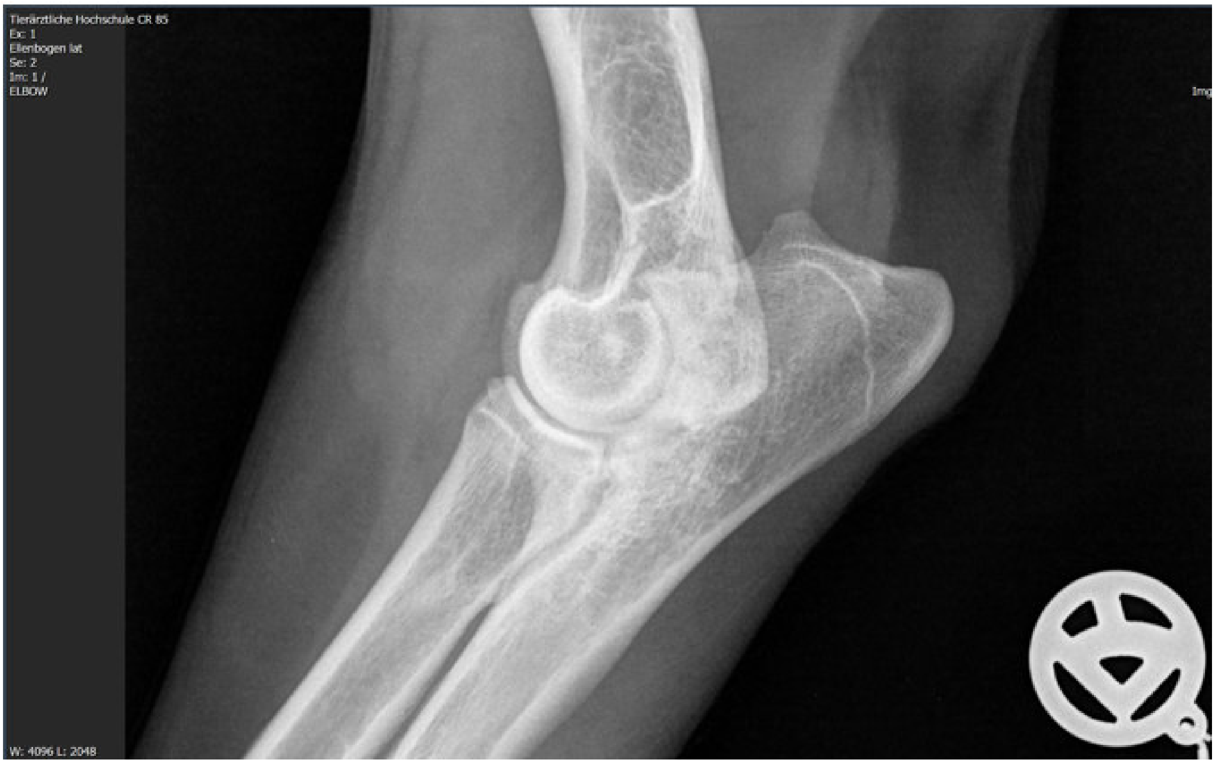

Radiograph of a right elbow in a mediolateral flexed view
Grade 1: Mild arthrosis - Presence of osteophytes < 2 mm high (shown in the measurement). Minor sclerosis of the base of the coronoid processes

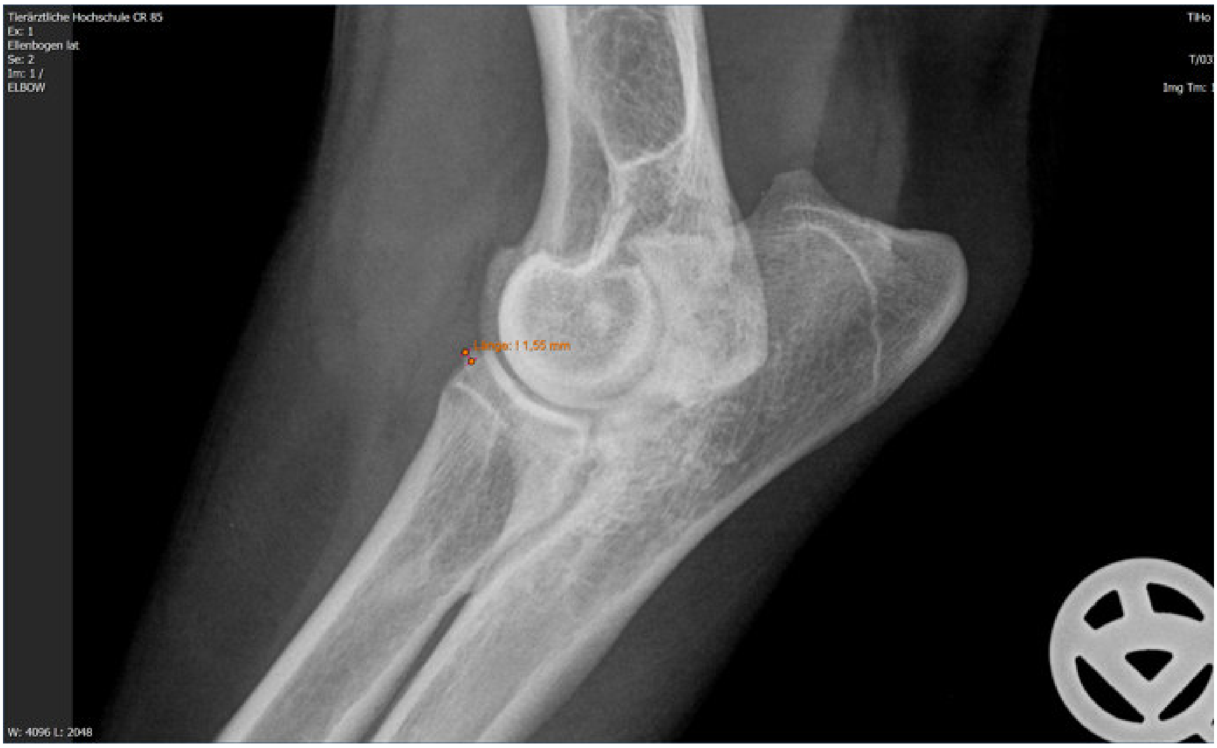

Radiograph of a right elbow in a craniocaudal view
Grade 1: Mild arthrosis - Presence of osteophytes < 2 mm high. Minor sclerosis of the base of the coronoid processes

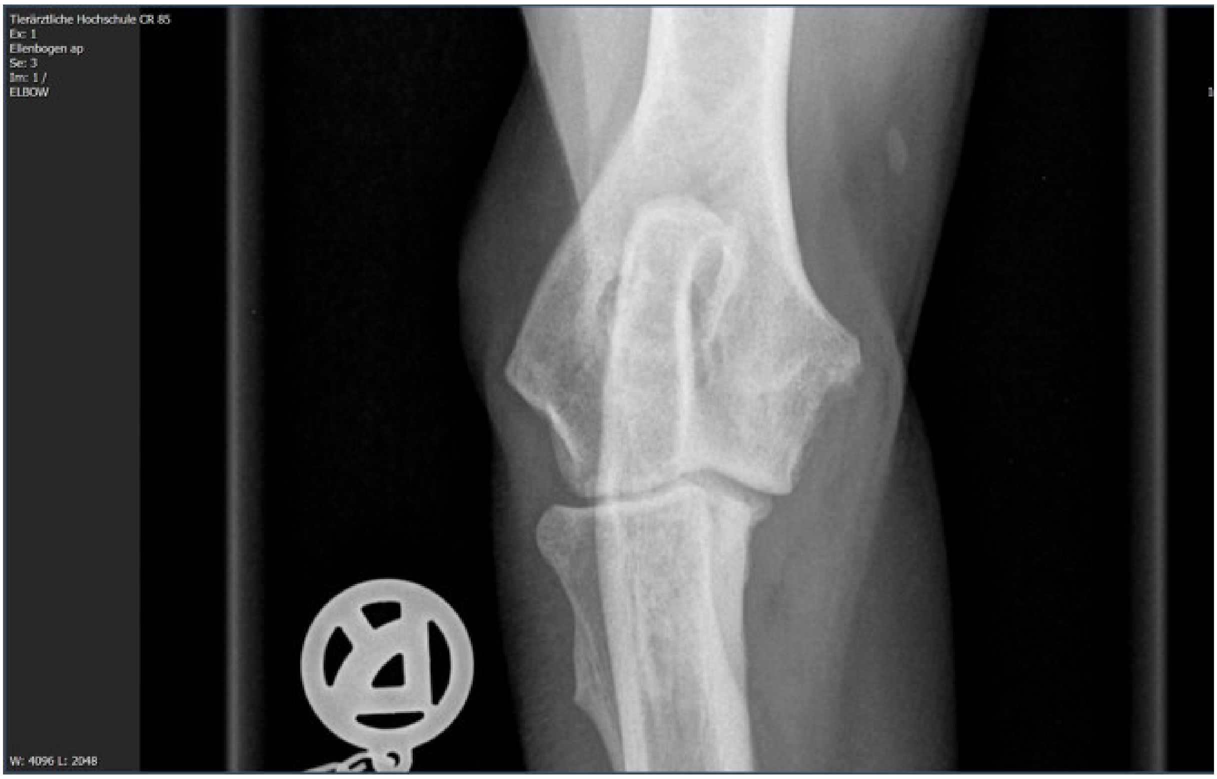

Radiograph of a right elbow in a craniocaudal view
Grade 1: Mild arthrosis - Presence of osteophytes < 2 mm high (shown in the measurement). Minor sclerosis of the base of the coronoid processes

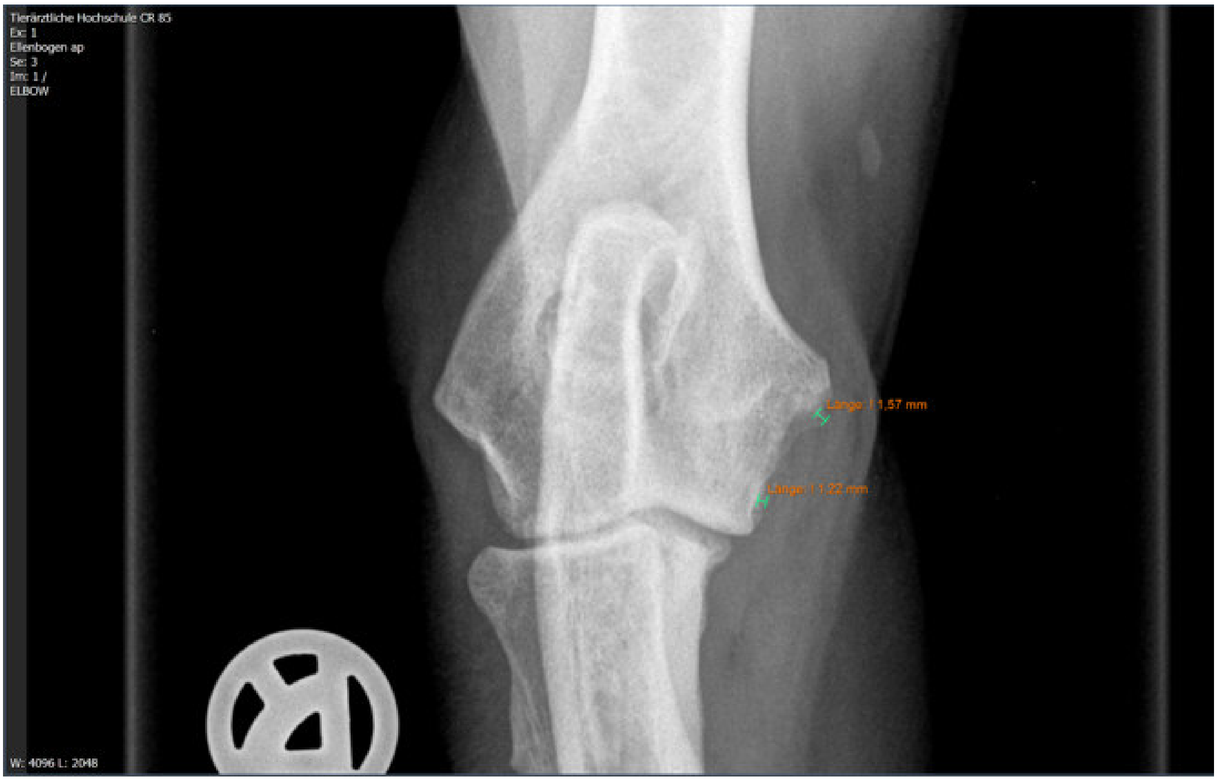

Modified Elbow Dysplasia Scoring – Grade 2

Radiograph of a left elbow in a mediolateral flexed view
Grade 2: Moderate arthrosis - Presence of osteophytes of 2 - 5 mm high. Obvious sclerosis of the base of the coronoid processes

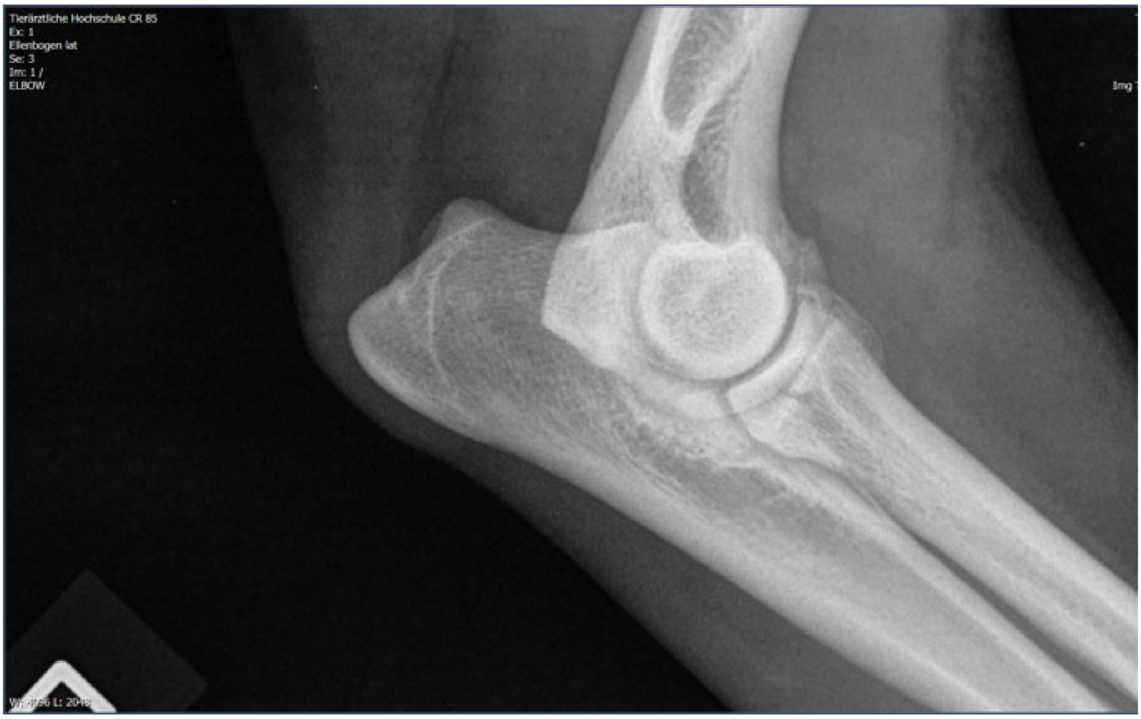

Radiograph of a left elbow in a mediolateral flexed view
Grade 2: Moderate arthrosis - Presence of osteophytes of 2 - 5 mm high (shown in the measurement). Obvious sclerosis of the base of the coronoid processes

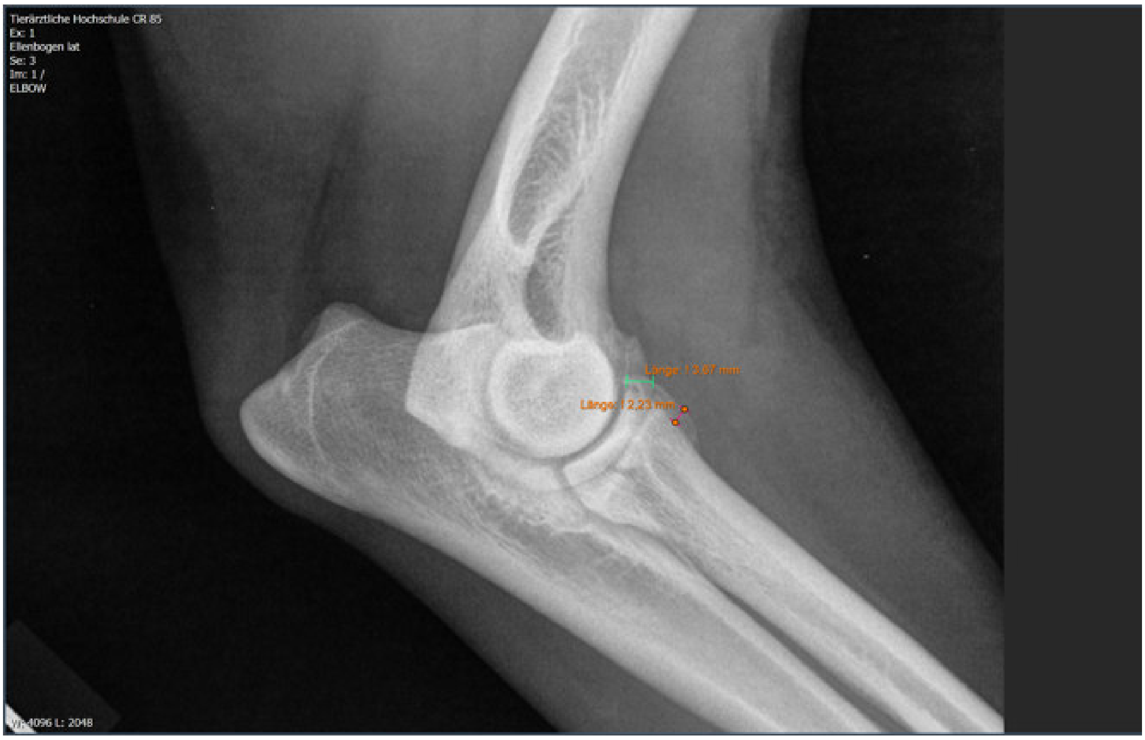

Radiograph of a left elbow in a craniocaudal view
Grade 2: Moderate arthrosis - Presence of osteophytes of 2 - 5 mm high. Obvious sclerosis of the base of the coronoid processes

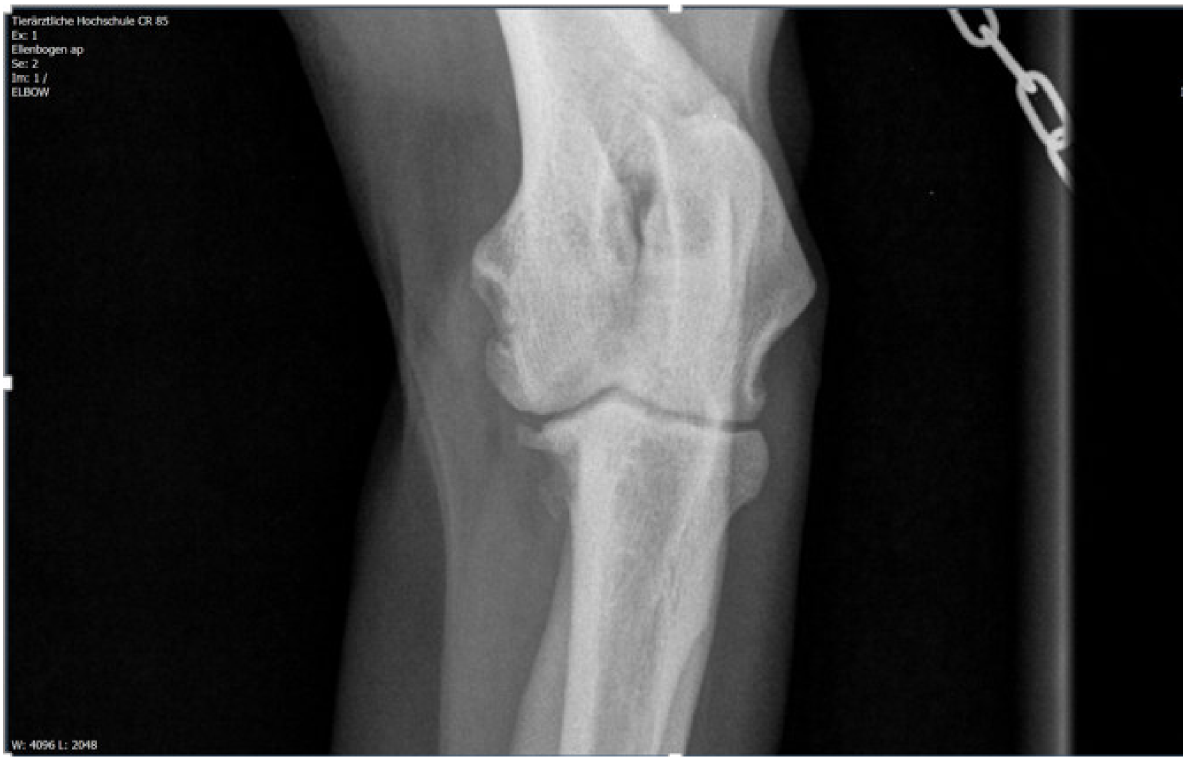

Radiograph of a left elbow in a craniocaudal view
Grade 2: Moderate arthrosis - Presence of osteophytes of 2 - 5 mm high (shown in the measurement). Obvious sclerosis of the base of the coronoid processes

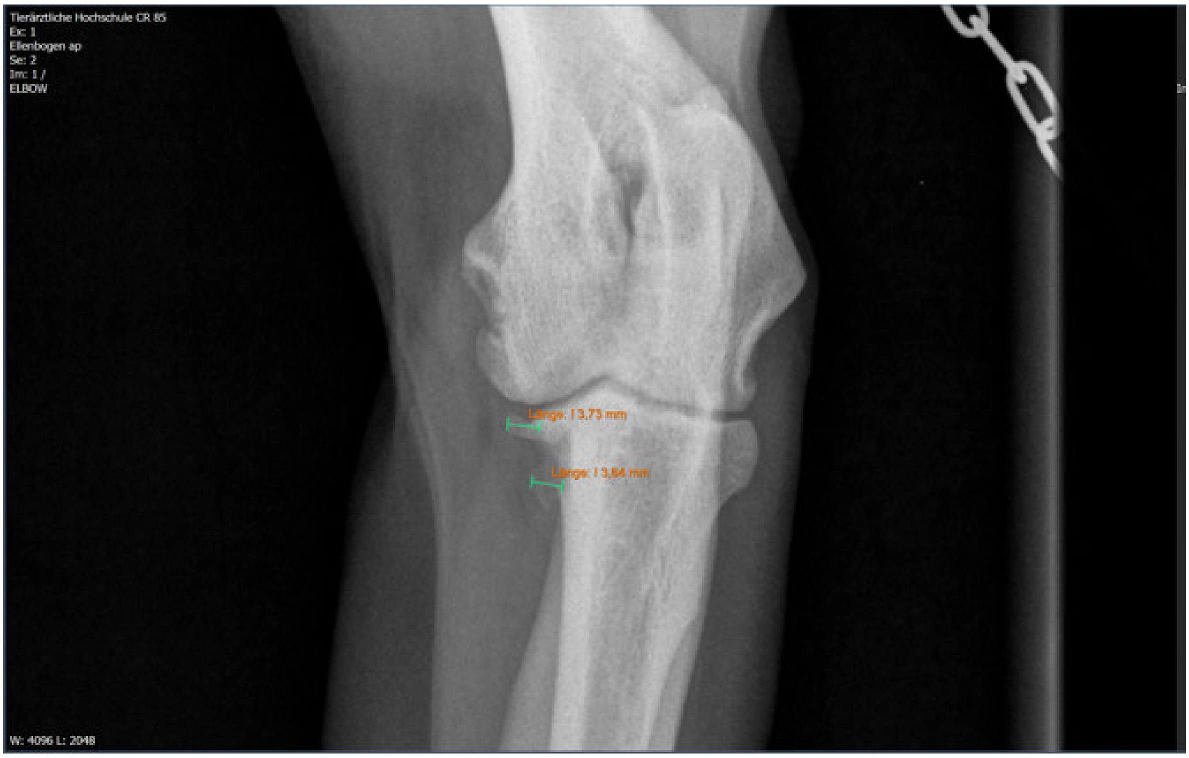

Modified Elbow Dysplasia Scoring – Grade 3
Radiograph of a right elbow in a mediolateral flexed view
Grade 3: Severe arthrosis - Presence of osteophytes of > 5 mm high.

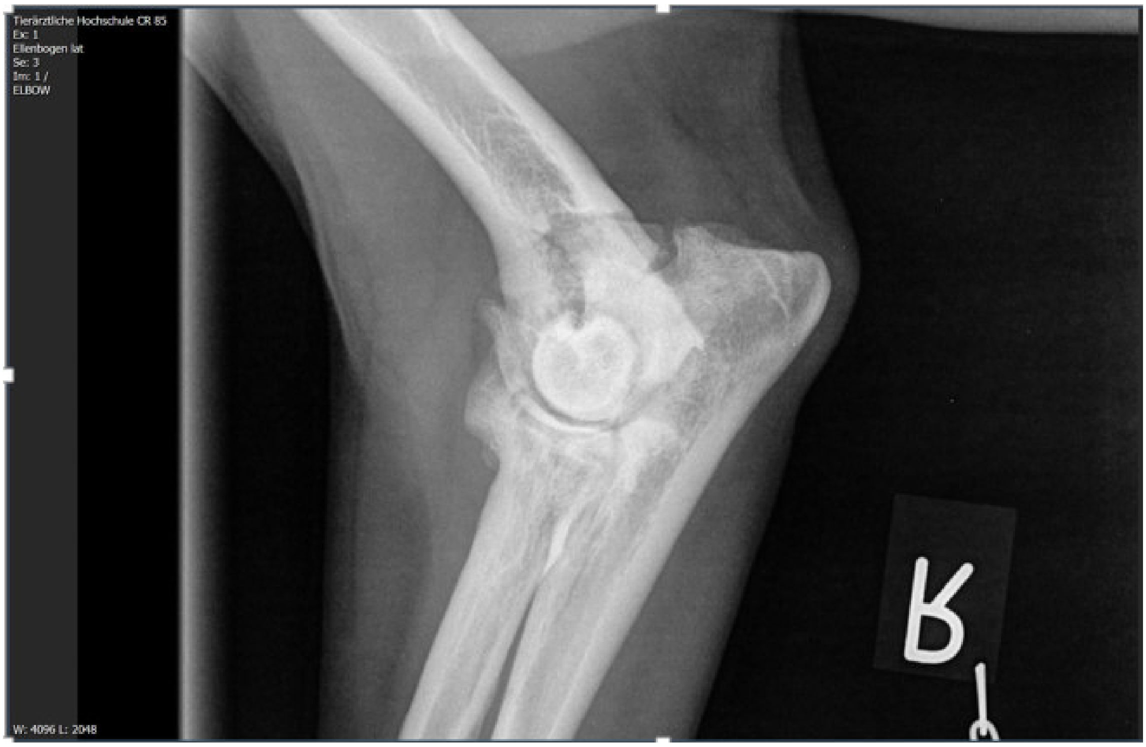

Radiograph of a right elbow in a mediolateral flexed view
Grade 3: Severe arthrosis - Presence of osteophytes of > 5 mm high (shown in the measurement).

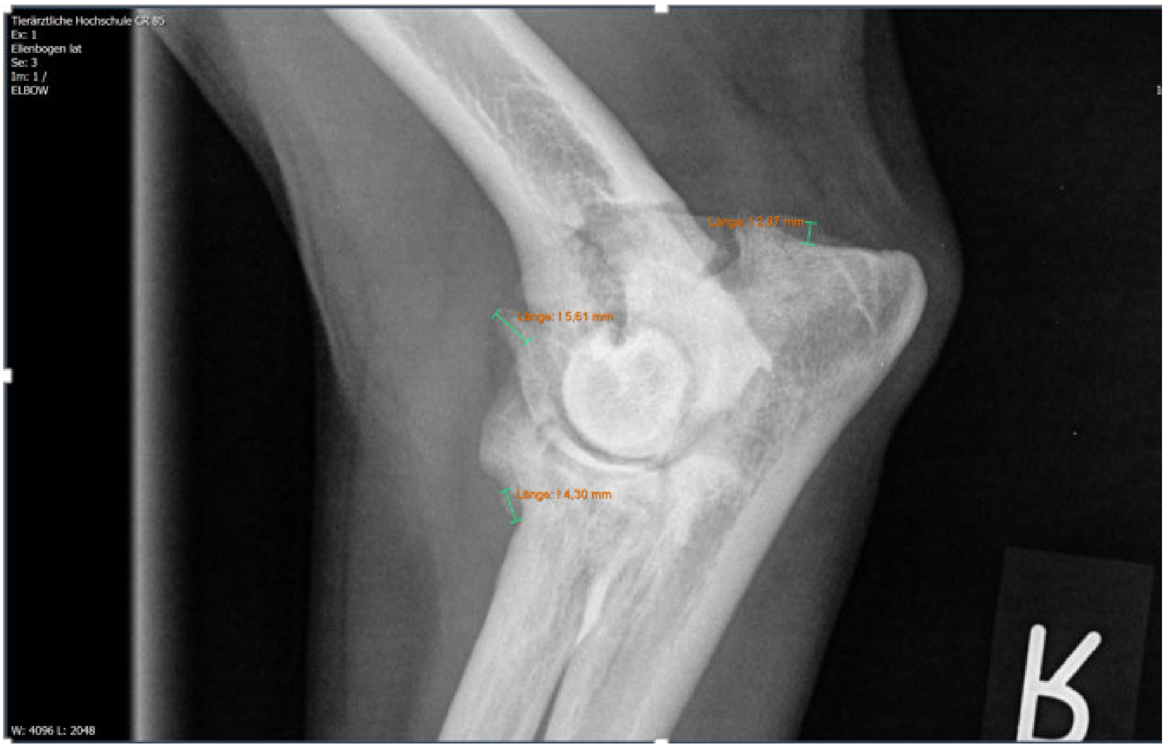

Radiograph of a right elbow in a craniocaudal view
Grade 3: Severe arthrosis - Presence of osteophytes of > 5 mm high.

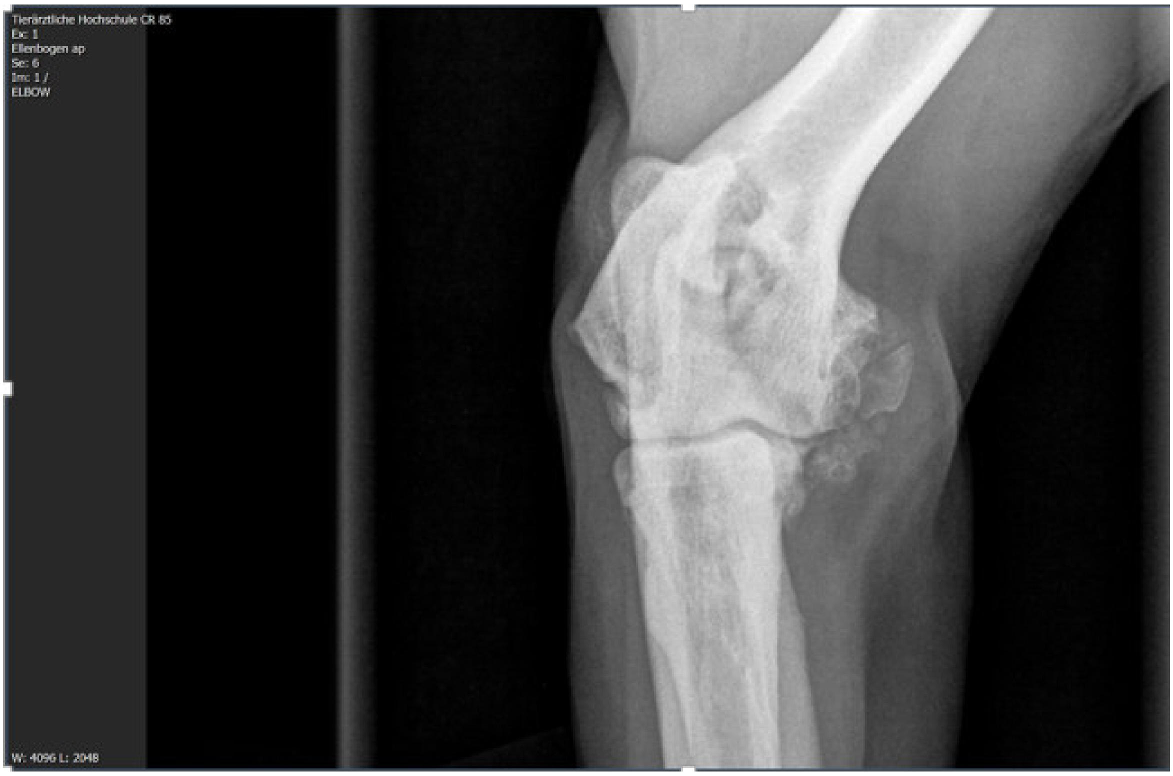

Radiograph of a right elbow in a craniocaudal view
Grade 3: Severe arthrosis - Presence of osteophytes of > 5 mm high (shown in the measurement).

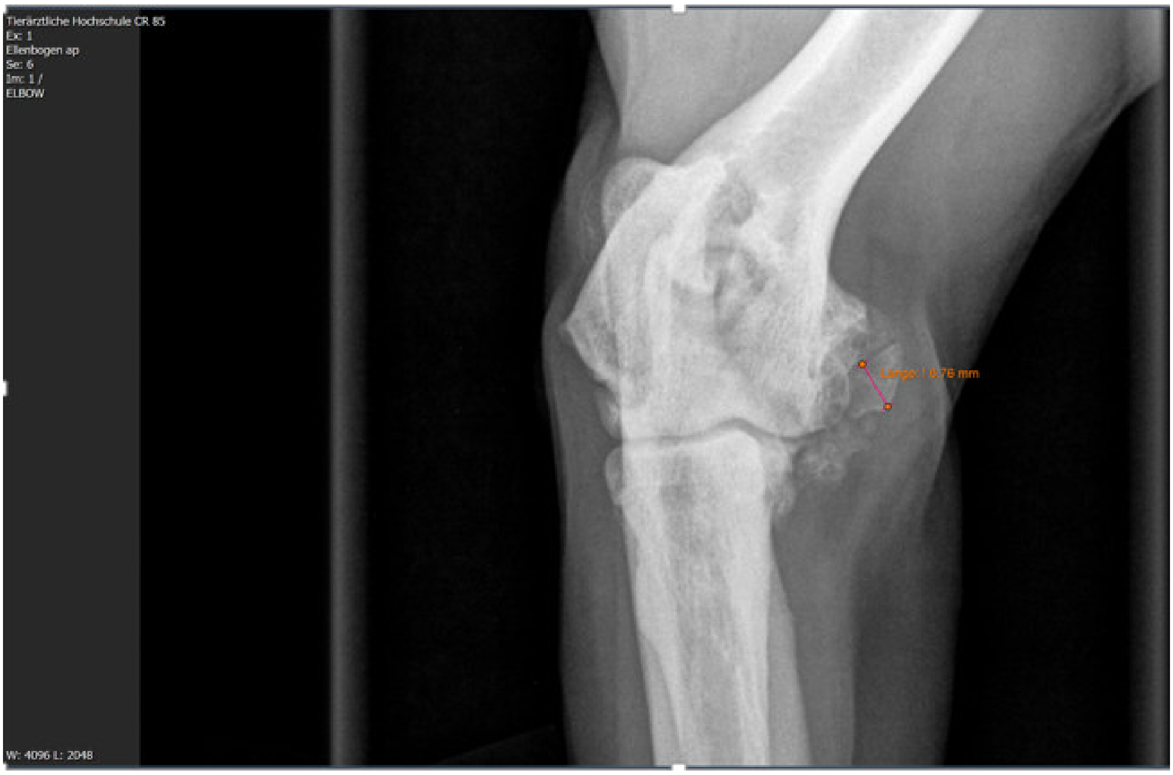

Table 3. Computed tomographic variables studied at the medial coronoid process (MCP) [16,27,28].

Type of pathology present at MCP	Type of fragmentation of the MCP	Fragment dislocation
1. Single fragment	1. Fragment or fissure along the radial incisure of the ulna	
2. Multiple fragments		
3. Fissures	2. Fragmentation affecting the MCP at the apex	1. Yes
4. Combination of lesions	3. Radial incisures – Tip fragment or fissure (Combination)	2. No
5. None of the above lesions		

Type of pathology present at MCP:

CT of a right elbow; Single fragment (arrow)

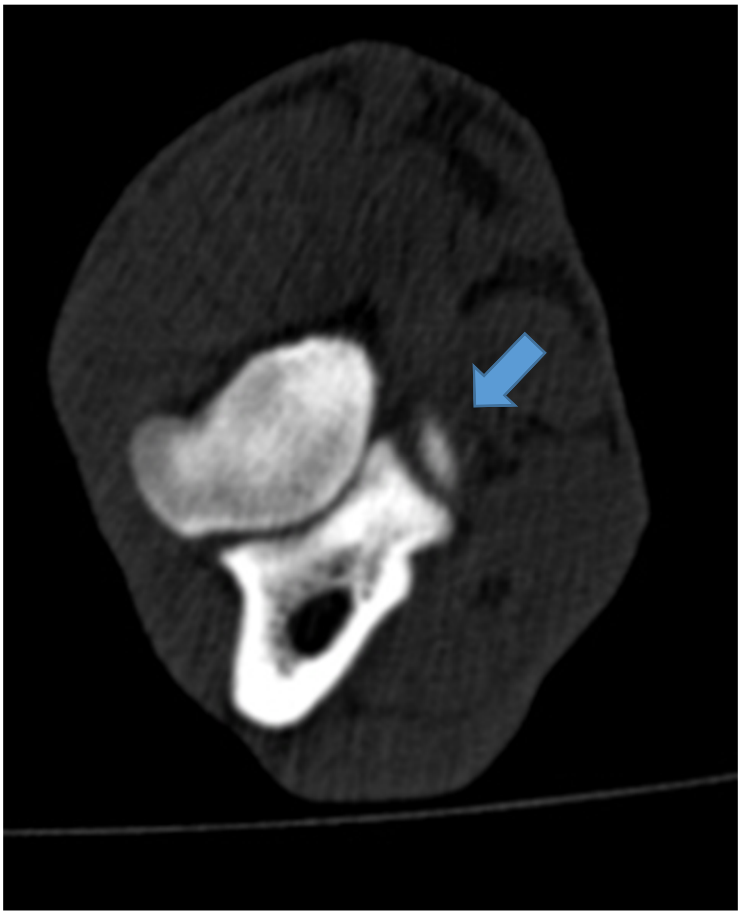

CT of a right elbow; multiple fragments (arrow)

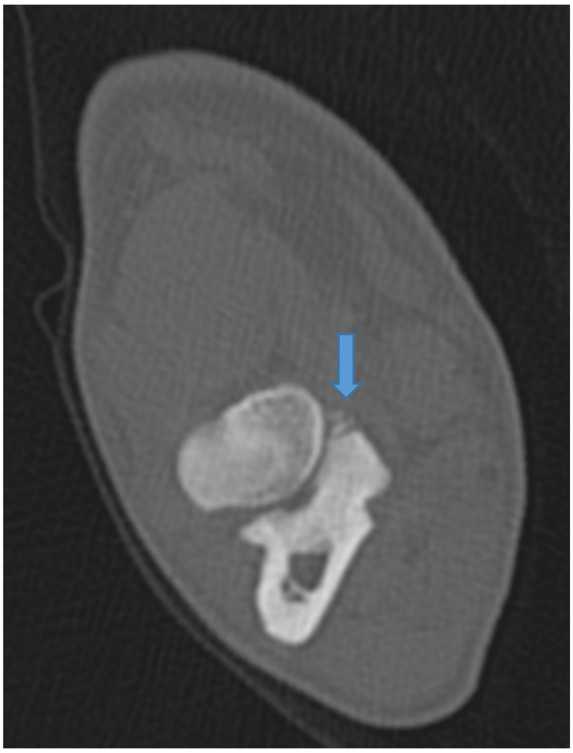

CT of a right elbow; Fissure at the tip of the medial coronoid process (arrow)

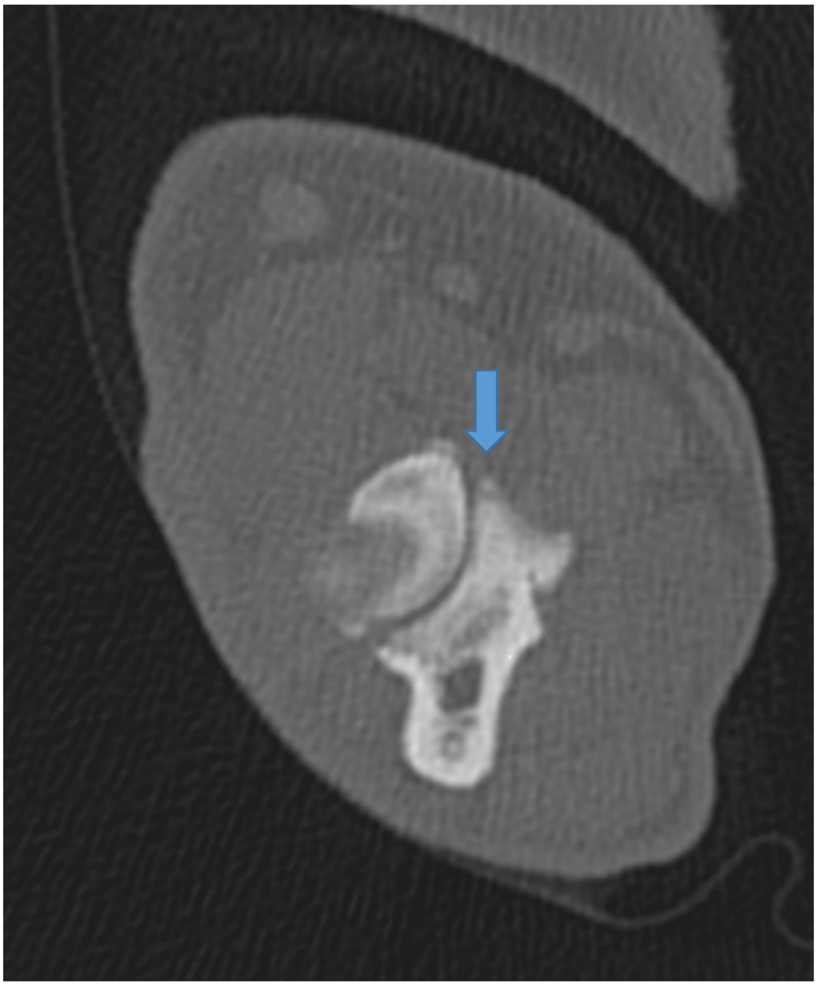

Type of fragmentation of the MCP:

CT of a right elbow; Fragment or fissure along the radial incisure of the ulna (arrow)

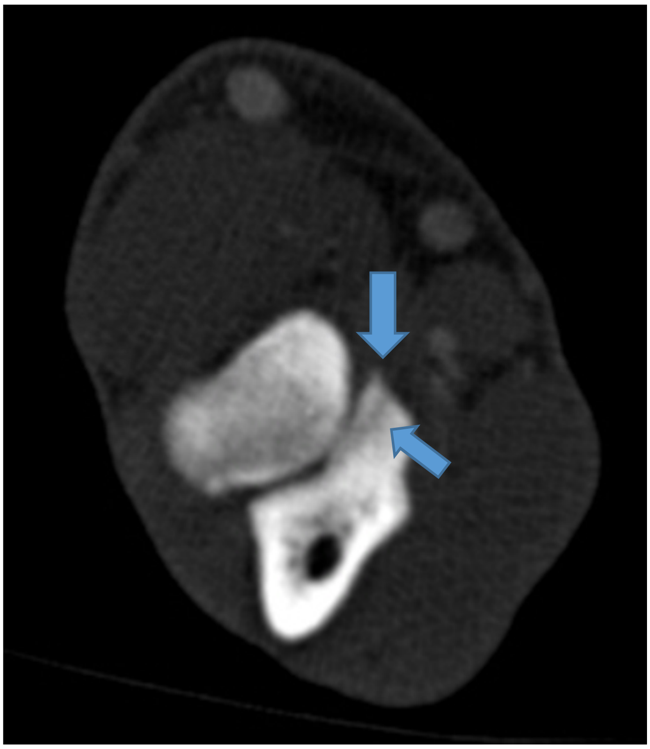

CT of a left elbow; Tip fragment (arrow)

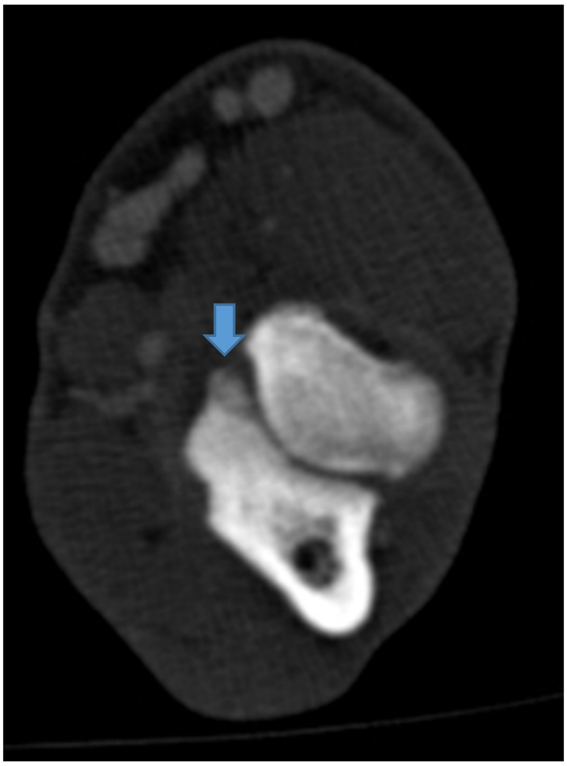

Fragment dislocation

CT of a left elbow; dislocated fragment (arrow)

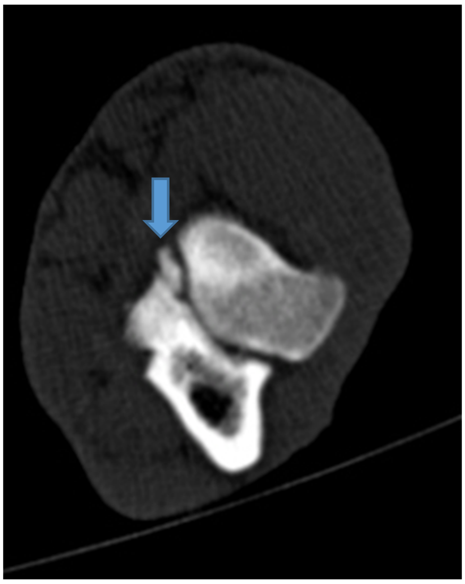

CT of a right elbow; non-dislocated fragment (arrow)

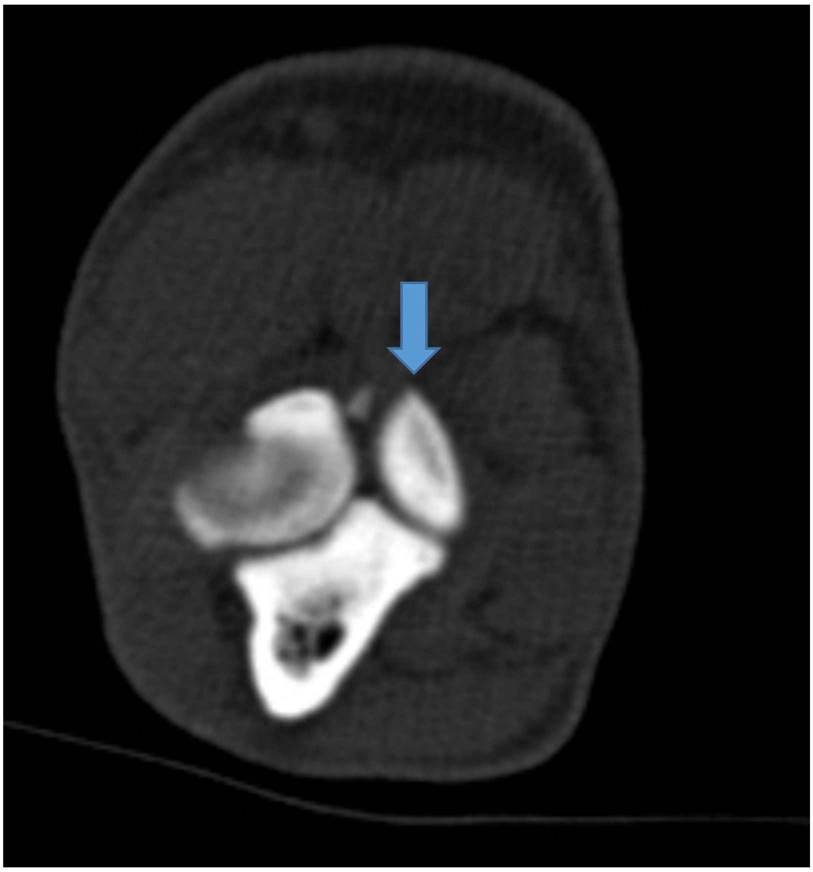

Supplement: Supplementary file 1 [file animals-13-03803-s001.zip › Provided figure for radiographic and CT images scoring.pdf]
