# Supplementary material for: Dogs with Bilateral Medial Coronoid Disease Can Be Clinically Sound after Unilateral Arthroscopic Fragment Removal—Preliminary Study
Source: Animals (Basel). 2023 Dec 9;13(24):3803. doi: 10.3390/ani13243803 (PMC10741036; doi:10.3390/ani13243803)
Supplement: Supplementary file 1 [file animals-13-03803-s001.zip › Supplementary file S1, English version.pdf]

University of Veterinary Medicine Foundation

Hannover

Clinic for small animals

Bünteweg 9

30559 Hanover

Questionnaire

Owner questionnaire for dogs with mobility problems/gait problems

Dear dog owners,

With your help, you enable us to obtain useful information about your dog and his condition, which contributes to our endeavours to treat painful and limiting diseases such as osteoarthritis, which is a consequence of elbow joint dysplasia.

It is important that you tick only one box per question from sections I + II + III + V, unless it is explicitly stated that several boxes can be ticked (e.g. question 4 under lifestyle habits/conduct).

For Section IV, the exact instructions for answering the questions are explained below.

If you have any concerns/difficulties about how to answer certain questions, please feel free to contact me or leave the answer open and we will discuss it together if you come to the clinic for a follow-up visit.

Thank you in advance!

Name of the owner: Click or tap here to enter text.

Telephone number: Click or tap here to enter text.

Name of the animal: Click or tap here to enter text.

Animal number (will be filled in by us): Click or tap here to enter text.

Sex of the dog: Click or tap here to enter text.

Date of birth animal: Click or tap here to enter text.

Breed of the dog: Click or tap here to enter text.

Date: Click or tap here to enter text.

Therapy of the elbow joint:

Arthroscopic/surgical: Right ☐ Left ☐

Conservative: Right ☐ Left ☐

(=painkillers, physiotherapy or similar)

Section I - Background to the animal:

1. How long has your animal been suffering from lameness/gait problems (before therapy)?

- Shorter than 6 months ☐
- 6 - 12 months ☐
- 12 - 24 months ☐
- 24 - 36 months ☐
- Longer than 36 months ☐

2. Has your pet been diagnosed with any other condition(s) (in addition to the orthopaedic problem)?

- No ☐
- Yes ☐
- If yes, please list these illnesses, if possible:

Click or tap here to enter text.

3. Is your pet currently receiving or has your pet received medication?

- No ☐

- If yes, ☐

→ Which medication: Click or tap here to enter text.

→ Dosage + frequency: Click or tap here to enter text.

→ When was the last dose administered? Click or tap here to enter text.

Section II - Lifestyle/Lifestyle:

1. How much exercise has your dog had on average per day in the last 7 days?

- 0 - 1 km ☐

- 1 - 2 km ☐

- 2 - 3 km ☐

- 3 - 4 km ☐

- More than 4 km ☐

2. How often was your dog walked on average per day in the last week?

- 0 ☐

- 1 ☐

- 2 ☐

- 3 ☐

- 4 ☐

- More often than 4 ☐

3. How is the walk/walk organised?

- Always on a leash ☐

- Mostly on a leash ☐

- Mostly without leash ☐

- Always without a leash ☐

- Mainly exercises ☐

4. Are there certain days of the week when your dog gets significantly more exercise than on others?

(You can also tick more than one box)

- Monday ☐
- Tuesday ☐
- Wednesday ☐
- Thursday ☐
- Friday ☐
- Saturday ☐
- Sunday ☐

5. On which surface/floor do most walks take place?

- In the grass ☐
- In the forest ☐
- On the road ☐
- Uneven terrain ☐

6. How is your dog walked during the walk?

- On the leash ☐
- Without leash ☐
- At the trot ☐
- Freewheel ☐

7. Who is the limiting factor when walking the dog?

- You ☐
- Your dog ☐

Section III - General mobility

1. How is your dog's general mobility?

- Very good ☐
- Good ☐
- Average ☐
- Poor ☐
- Very bad ☐

2. How active is your dog in general?

- Extremely active ☐
- Active ☐
- Moderately active ☐
- Less active ☐
- Not active at all ☐

3. How active is your dog during walks?

- Extremely active ☐
- Very active ☐
- Moderately active ☐
- Not very active ☐
- Not active at all ☐

4. How interested/motivated is your dog to go for a walk?

- Extremely interested/motivated ☐
- Very interested/motivated ☐
- Moderately interested/motivated ☐
- Less interested/motivated ☐
- Not at all interested/motivated ☐

5. How would you rate your dog's trainability and ability to go for a walk?

- Very good ☐
- Good ☐
- Mediocre ☐
- Bad ☐
- Very bad ☐

6. How often does your dog rest during the walk (stops, sits down)?

- Never ☐
- Hardly ☐
- Occasionally ☐
- Frequently/regularly ☐
- Very common ☐

<u>Section IV - Special mobility</u>

This section deals specifically with the comparison between the two diseased forelimbs and the therapeutic success of the surgical/conservative treatment. This means that each forelimb/elbow joint is <u>assessed individually</u> and <u>independently of each other</u>. There are therefore two possible answers if there is a difference. Please complete this section of the questionnaire using the following key:

R = right forelimb/elbow joint

L = left forelimb/elbow joint

B = both forelimbs, if there is no difference between right and left limb

1. How impaired is your dog due to his/her lameness?

- Not affected at all R☐ L☐ B☐
- Slightly impaired R☐ L☐ B☐
- Moderately impaired R☐ L☐ B☐
- Severely impaired R☐ L☐ B☐
- Extremely impaired R☐ L☐ B☐

2. What effect does cold, damp weather have on your dog's lameness?

- No effect R☐ L☐ B☐

- Mild effect R☐ L☐ B☐

- Moderate effect R☐ L☐ B☐

- Strong effect R☐ L☐ B☐

- Very strong effect R☐ L☐ B☐

3. What degree of movement restriction does your dog show on the affected leg(s) after a "nap"/rest?

- No restriction of movement R☐ L☐ B☐

- Mild restriction of movement R☐ L☐ B☐

- Moderate restriction of movement R☐ L☐ B☐

- Severe restriction of movement R☐ L☐ B☐

- Extreme restriction of movement R☐ L☐ B☐

4. What is the overall effect of training/walking on your dog's lameness?

Please place an additional cross here for positive or negative overall impact.

- No effect R☐ L☐ B☐

- Mild effect R☐ L☐ B☐

- Moderate impact - Strong impact R☐ L☐ B☐

- Very strong effect R☐ L☐ B☐

- Positive overall effect R☐ L☐ B☐

- Negative overall effect R☐ L☐ B☐

5. What effect does cold, damp weather have on walking your dog (in terms of gait)?

- No effect R☐ L☐ B☐

- Moderate impact R☐ L☐ B☐

- Strong impact R☐ L☐ B☐

- Very strong effect R☐ L☐ B☐

6. What degree of movement restriction does your dog show on the affected leg(s) during an exercise after a "nap"/rest?

- No restriction of movement R☐ L☐ B☐

- Mild restriction of movement R☐ L☐ B☐

- Moderate restriction of movement R☐ L☐ B☐

- Severe restriction of movement R☐ L☐ B☐

- Extreme restriction of movement R☐ L☐ B☐

7. What effect does your dog's lameness have when you walk him?

Please place an additional cross here for positive or negative overall impact.

- No effect R☐ L☐ B☐

- Mild effect R☐ L☐ B☐

- Moderate impact R☐ L☐ B☐

- Strong impact R☐ L☐ B☐

- Very strong effect R☐ L☐ B☐

- Positive overall effect R☐ L☐ B☐

- Negative overall effect R☐ L☐ B☐

Section V - Additional questions

1. Has your pet been diagnosed with another orthopaedic condition (in addition to the bilateral elbow joint problems)?

- No ☐

- Yes ☐

- If yes, the following: Click here to enter text.

2. What is the intended use of the dog?

- Family dog ☐

- Sport/Competitions ☐

- Hunting ☐

- Other: Click here to enter text.

3. Was there an improvement/deterioration after the therapy? If yes, after how many weeks/months?

Surgically treated limb:

- Improvement ☐

- Deterioration ☐

- After which period: Click here to enter text.

- First deterioration, then improvement ☐

- First improvement, then deterioration ☐

Conservative (painkillers, physiotherapy, etc.):

- Improvement ☐

- Deterioration ☐

- After which period: Click here to enter text.

- First deterioration, then improvement ☐

- First improvement, then deterioration ☐

4. What rehabilitation measures were taken after the operation on the surgically treated leg?

- None ☐

- Physiotherapy ☐

- Water treadmill ☐

- Other: Click here to enter text.

5. How was the conservatively treated leg treated?

- Not at all ☐

- Physiotherapy ☐

- Water treadmill ☐

- Other: Click here to enter text.

6. is your dog in pain or does your dog have problems lying down?

- No ☐

- Yes ☐

- If yes, which limb is most affected: Click here to enter text.

Thank you very much for answering this questionnaire!
