# Supplementary material for: Dogs with Bilateral Medial Coronoid Disease Can Be Clinically Sound after Unilateral Arthroscopic Fragment Removal—Preliminary Study
Source: Animals (Basel). 2023 Dec 9;13(24):3803. doi: 10.3390/ani13243803 (PMC10741036; doi:10.3390/ani13243803)
Supplement: Supplementary file 1 [file animals-13-03803-s001.zip › Supplementary file S1.pdf]

Stiftung Tierärztliche Hochschule

Hannover

Klinik für Kleintiere

Bünteweg 9

30559 Hannover

Fragebogen

Besitzerfragebogen für Hunde mit Mobilitätsproblemen/Gangproblemen

Sehr geehrte Hundebesitzer,

durch Ihre Hilfe ermöglichen Sie uns nützliche Informationen über Ihren Hund bzw. seine Erkrankung zu erfahren, was dazu beiträgt, unser Bestreben, schmerzhafte und einschränkende Krankheiten wie beispielsweise die Arthrose, was unter anderem eine Folge der Ellenbogengelenksdysplasie ist, noch besser behandeln zu können.

Es ist wichtig, dass Sie pro Frage aus Abschnitt I + II + III + V nur ein Kästchen ankreuzen, außer es steht ausdrücklich dabei, dass mehrere Kästchen angekreuzt werden können (z.B. Frage 4 unter Lebensgewohnheiten/-führung).

Für den Abschnitt IV wird die genaue Anweisung für die Beantwortung der Fragen unten erläutert.

Falls Sie irgendwelche Bedenken/Schwierigkeiten haben, wie Sie bestimmte Fragen beantworten sollen, kontaktieren Sie mich gerne oder lassen Sie die Antwort offen und wir besprechen diese gemeinsam, falls Sie zu einer Nachkontrolle in die Klinik kommen.

Ein herzliches Dankeschön bereits im Voraus!

Name des Besitzers: Klicken oder tippen Sie hier, um Text einzugeben.

Telefonnummer: Klicken oder tippen Sie hier, um Text einzugeben.

Name des Tieres: Klicken oder tippen Sie hier, um Text einzugeben.

Tiernummer (wird von uns ausgefüllt): Klicken oder tippen Sie hier, um Text einzugeben.

Geschlecht des Hundes: Klicken oder tippen Sie hier, um Text einzugeben.

Geburtsdatum Tier: Klicken oder tippen Sie hier, um Text einzugeben.

Rasse des Hundes: Klicken oder tippen Sie hier, um Text einzugeben.

Datum: Klicken oder tippen Sie hier, um Text einzugeben.

Therapie des Ellenbogengelenkes:

Arthroskopisch/chirurgisch: Rechts ☐ Links ☐

Konservativ: Rechts ☐ Links ☐

(=Schmerzmittel, Physiotherapie o.ä.)

Abschnitt I – Hintergrund zum Tier:

1. Wie lange litt Ihr Tier schon unter der Lahmheit/Gangproblemen (vor der Therapie)?

- kürzer als 6 Monate ☐
- 6 – 12 Monate ☐
- 12 – 24 Monate ☐
- 24 – 36 Monate ☐
- länger als 36 Monate ☐

2. Wurde bei Ihrem Tier eine/mehrere weitere Erkrankung/en diagnostiziert (zusätzlich zu dem orthopädischen Problem)?

- Nein ☐
- Ja ☐
- Falls ja, bitte listen Sie diese Erkrankungen, wenn möglich, auf:

Klicken oder tippen Sie hier, um Text einzugeben.

3. Erhält Ihr Tier aktuell bzw. bekam Ihr Tier Medikamente?

- Nein ☐
- Falls ja, ☐

→ Welche Medikamente: Klicken oder tippen Sie hier, um Text einzugeben.

→ Dosierung + Häufigkeit: Klicken oder tippen Sie hier, um Text einzugeben.

→ Wann wurde die letzte Dosis verabreicht?: Klicken oder tippen Sie hier, um Text einzugeben.

Abschnitt II - Lebensgewohnheiten/Lebensführung:

1. Wie viel Bewegung hatte Ihr Hund durchschnittlich pro Tag in den letzten 7 Tagen?

- 0 – 1 km ☐
- 1 – 2 km ☐
- 2 – 3 km ☐
- 3 – 4 km ☐
- mehr als 4 km ☐

2. Wie häufig wurde mit Ihrem Hund in der letzten Woche durchschnittlich pro Tag spazieren gegangen?

- 0 ☐
- 1 ☐
- 2 ☐
- 3 ☐
- 4 ☐
- öfter als 4 ☐

3. Wie ist der Spaziergang/das Gassi gehen gestaltet?

- immer an der Leine ☐
- meistens an der Leine ☐
- meistens ohne Leine ☐

- immer ohne Leine ☐

- überwiegend Übungen ☐

4. Gibt es bestimmte Tage in der Woche, an denen Ihr Hund deutlich mehr Bewegung hat als an anderen?

(Es können auch mehrere Sachen angekreuzt werden)

- Montag ☐

- Dienstag ☐

- Mittwoch ☐

- Donnerstag ☐

- Freitag ☐

- Samstag ☐

- Sonntag ☐

5. Auf welchem Untergrund/Boden finden die meisten Gassi Gänge statt?

- Im Gras ☐

- Im Wald ☐

- Auf der Straße ☐

- Unebenes Gelände ☐

6. Wie wird Ihr Hund während des Gassi Gehens geführt?

- An der Leine ☐

- Ohne Leine ☐

- Im Trab ☐

- Freilauf ☐

7. Wer ist der einschränkende Faktor beim Gassi gehen?

- Sie ☐

- Ihr Hund ☐

Abschnitt III – Allgemeine Mobilität

1. Wie ist die allgemeine Bewegungsfähigkeit Ihres Hundes?

- Sehr gut ☐
- Gut ☐
- Durchschnittlich ☐
- Mangelhaft ☐
- Sehr schlecht ☐

2. Wie aktiv ist Ihr Hund generell?

- Überaus aktiv ☐
- Aktiv ☐
- Mittelmäßig aktiv ☐
- Weniger aktiv ☐
- Überhaupt nicht aktiv ☐

3. Wie aktiv ist Ihr Hund während der Gassi Gänge?

- Extrem aktiv ☐
- Sehr aktiv ☐
- Mittelmäßig aktiv ☐
- Nicht sehr aktiv ☐
- Überhaupt nicht aktiv ☐

4. Wie interessiert/motiviert ist Ihr Hund daran Gassi zu gehen?

- Extrem interessiert/motiviert ☐
- Sehr interessiert/motiviert ☐
- Mittelmäßig interessiert/motiviert ☐
- Weniger interessiert/motiviert ☐
- Überhaupt nicht interessiert/motiviert ☐

5. Wie schätzen Sie die Trainingsfähigkeit Ihres Hundes, sowie seine Fähigkeit Gassi zu gehen ein?

- Sehr gut ☐
- Gut ☐
- Mittelmäßig ☐
- Schlecht ☐
- Sehr schlecht ☐

6. Wie häufig ruht sich Ihr Hund während des Gassi Gehens aus (stoppt, setzt sich hin)?

- Nie ☐
- Kaum ☐
- Gelegentlich ☐
- Häufig/regelmäßig ☐
- Sehr häufig ☐

<u>Abschnitt IV – Spezielle Mobilität</u>

In diesem Abschnitt geht es speziell um den Vergleich zwischen den beiden erkrankten Vordergliedmaßen und den Therapieerfolg durch die chirurgische/konservative Behandlung. Das heißt, jede/s Vordergliedmaße/Ellenbogengelenk wird <u>einzeln</u> und <u>unabhängig voneinander bewertet</u>. Somit gibt es zwei Antwortmöglichkeiten, wenn ein Unterschied vorhanden sein sollte. Füllen Sie bitte diesen Abschnitt des Fragebogens unter Verwendung des folgenden Schlüssels aus:

R = rechte Vordergliedmaße/Ellenbogengelenk

L = linke Vordergliedmaße/Ellenbogengelenk

B = beide Vordergliedmaßen, wenn es keinen Unterschied zwischen Rechter und Linker gibt

1. Wie beeinträchtigt ist Ihr Hund aufgrund seiner/ihrer Lahmheit?

- Überhaupt nicht beeinträchtigt R☐ L☐ B☐
- Leicht beeinträchtigt R☐ L☐ B☐
- Mittelmäßig beeinträchtigt R☐ L☐ B☐
- Stark beeinträchtigt R☐ L☐ B☐
- Extrem beeinträchtigt R☐ L☐ B☐

2. Welchen Effekt hat kaltes, feuchtes Wetter auf die Lahmheit Ihres Hundes?

- Keinen Effekt R☐ L☐ B☐
- Milden Effekt R☐ L☐ B☐
- Moderaten Effekt R☐ L☐ B☐
- Starken Effekt R☐ L☐ B☐
- Sehr starken Effekt R☐ L☐ B☐

3. Was für ein Ausmaß an Bewegungseinschränkung zeigt Ihr Hund am betroffenen Bein/den betroffenen Beinen nach einem „Nickerchen“/Ruhepause?

- Keine Bewegungseinschränkung R☐ L☐ B☐
- Milde Bewegungseinschränkung R☐ L☐ B☐
- Mittelmäßige Bewegungseinschränkung R☐ L☐ B☐
- Starke Bewegungseinschränkung R☐ L☐ B☐
- Extreme Bewegungseinschränkung R☐ L☐ B☐

4. Welche Gesamtauswirkung hat das Training/Gassi Gehen auf die Lahmheit Ihres Hundes?

Hier bitte ein zusätzliches Kreuz für positive bzw. negative Gesamtauswirkung setzen.

- Keine Auswirkung R☐ L☐ B☐
- Milde Auswirkung R☐ L☐ B☐
- Mittelmäßige Auswirkung - Starke Auswirkung R☐ L☐ B☐
- Sehr starke Auswirkung R☐ L☐ B☐
- positive Gesamtauswirkung R☐ L☐ B☐
- negative Gesamtauswirkung R☐ L☐ B☐

5. Was für eine Auswirkung hat kaltes, feuchtes Wetter auf das Gassi Gehen (bezogen auf das Gangbild) Ihres Hundes?

- Keine Auswirkung R☐ L☐ B☐
- Mittelmäßige Auswirkung R☐ L☐ B☐
- Starke Auswirkung R☐ L☐ B☐
- Sehr starke Auswirkung R☐ L☐ B☐

6. Was für ein Ausmaß an Bewegungseinschränkung zeigt Ihr Hund am betroffenen Bein/den betroffenen Beinen während einer Übung nach einem „Nickerchen“/Ruhepause?

- Keine Bewegungseinschränkung	R☐	L☐	B☐
- milde Bewegungseinschränkung	R☐	L☐	B☐
- mittelmäßige Bewegungseinschränkung	R☐	L☐	B☐
- starke Bewegungseinschränkung	R☐	L☐	B☐
- extreme Bewegungseinschränkung	R☐	L☐	B☐

7. Welche Auswirkung hat die Lahmheit Ihres Hundes während des Gassi Gehens?

Hier bitte ein zusätzliches Kreuz für positive bzw. negative Gesamtauswirkung setzen.

- Keine Auswirkung	R☐	L☐	B☐
- Milde Auswirkung	R☐	L☐	B☐
- Mittelmäßige Auswirkung	R☐	L☐	B☐
- Starke Auswirkung	R☐	L☐	B☐
- Sehr starke Auswirkung	R☐	L☐	B☐
- positive Gesamtauswirkung	R☐	L☐	B☐
- negative Gesamtauswirkung	R☐	L☐	B☐

Abschnitt V – Zusätzliche Fragen

1. Wurde bei Ihrem Tier (neben den beidseitigen Ellenbogengelenksproblemen) eine weitere orthopädische Erkrankung diagnostiziert?

- Nein ☐

- Ja ☐

- Falls ja, folgende: Click here to enter text.

2. Was ist der Verwendungszweck des Hundes?

- Familienhund ☐

- Sport/Wettkämpfe ☐

- Jagd ☐

- Andere: Click here to enter text.

3. Trat nach der Therapie eine Verbesserung/Verschlechterung ein? Falls ja, nach wie viel Wochen/Monaten?

Chirurgisch therapierte Gliedmaße:

- Verbesserung ☐

- Verschlechterung ☐

- Nach welchem Zeitraum: Click here to enter text.

- Erst Verschlechterung, dann Verbesserung ☐

- Erst Verbesserung, dann Verschlechterung ☐

Konservativ (Schmerzmittel, Physiotherapie o.ä.):

- Verbesserung ☐

- Verschlechterung ☐

- Nach welchem Zeitraum: Click here to enter text.

- Erst Verschlechterung, dann Verbesserung ☐

- Erst Verbesserung, dann Verschlechterung ☐

4. Welche Rehabilitationsmaßnahmen wurden nach der Operation am chirurgisch therapierten Bein vorgenommen?

- Keine ☐

- Physiotherapie ☐

- Wasserlaufband ☐

- Andere: Click here to enter text.

5. Wie wurde das konservativ behandelte Bein therapiert?

- Gar nicht ☐

- Physiotherapie ☐

- Wasserlaufband ☐

- Andere: Click here to enter text.

6. Zeigt Ihr Hund Schmerzen bzw. hat Ihr Hund Probleme sich hinzulegen?

- Nein ☐

- Ja ☐

- Falls ja, welche Gliedmaße ist am stärksten betroffen: Click here to enter text.

Vielen herzlichen Dank für die Beantwortung dieses Fragebogens!
